# Stochastic Drift in Mitochondrial DNA Point Mutations: A Novel Perspective *Ex Silico*

**DOI:** 10.1371/journal.pcbi.1000572

**Published:** 2009-11-20

**Authors:** Suresh Kumar Poovathingal, Jan Gruber, Barry Halliwell, Rudiyanto Gunawan

**Affiliations:** 1 Department of Chemical and Biomolecular Engineering, National University of Singapore, Singapore; 2 Department of Biochemistry, Neurobiology and Ageing Program, Centre for Life Science (CeLS), Singapore; Johns Hopkins University, United States of America

## Abstract

The mitochondrial free radical theory of aging (mFRTA) implicates Reactive Oxygen Species (ROS)-induced mutations of mitochondrial DNA (mtDNA) as a major cause of aging. However, fifty years after its inception, several of its premises are intensely debated. Much of this uncertainty is due to the large range of values in the reported experimental data, for example on oxidative damage and mutational burden in mtDNA. This is in part due to limitations with available measurement technologies. Here we show that sample preparations in some assays necessitating high dilution of DNA (single molecule level) may introduce significant statistical variability. Adding to this complexity is the intrinsically stochastic nature of cellular processes, which manifests in cells from the same tissue harboring varying mutation load. In conjunction, these random elements make the determination of the underlying mutation dynamics extremely challenging. Our *in silico* stochastic study reveals the effect of coupling the experimental variability and the intrinsic stochasticity of aging process in some of the reported experimental data. We also show that the stochastic nature of a *de novo* point mutation generated during embryonic development is a major contributor of different mutation burdens in the individuals of mouse population. Analysis of simulation results leads to several new insights on the relevance of mutation stochasticity in the context of dividing tissues and the plausibility of ROS ”vicious cycle” hypothesis.

## Author Summary

Aging is characterized by a systemic decline of an organism's capacity in responding to internal and external stresses, leading to increased mortality. The mitochondrial Free Radical Theory of Aging (mFRTA) attributes this decline to the accumulation of damages, in the form of mitochondrial DNA (mtDNA) mutations, caused by free radical byproducts of metabolism. However, there is still a great deal of uncertainty with this theory due to the difficulties in quantifying mtDNA mutation burden. In this modeling study, we have shown that a random drift in mtDNA point mutation during life, in combination with the experimental sampling can explain the variability seen in some of the reported experimental data. Particularly, we found that while the average mutation increases in a linear fashion, the variability in the mutation load data increases over time, and thus a low number of data replicates can often lead to a deceptive inference of the mutation burden dynamics. The model also predicted a significant contribution from the embryonic developmental phase to the accumulation of mtDNA mutation burden. Furthermore, the model revealed that the replication rate of mtDNA is a major determinant of new mutations during development and in fast-dividing tissues.

## Introduction

**Figure 1:**
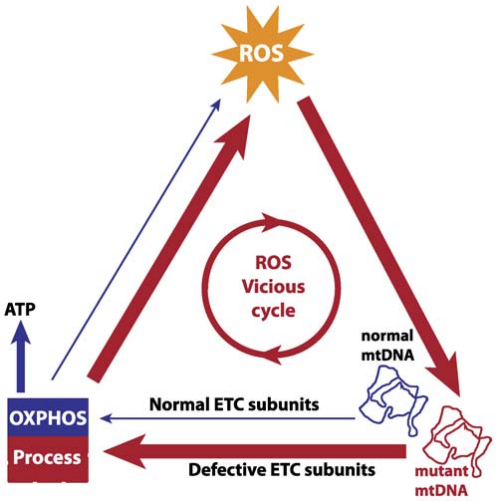
Mitochondrial ROS vicious cycle. A putative positive feedback mechanism between mtDNA and ROS is based on the hypothesis that ROS-induced damaged mtDNA produce defective components of the ETC, thereby increasing electron leakage in the OXPHOS process and ROS production. The vicious cycle is expected to give an exponential expansion of mtDNA mutations over time, which eventually causes the loss of mitochondrial function in generating ATP.

Mitochondria are the main energy producing organelles present in eukaryotic cells. Mitochondria are the only organelles aside from the nucleus which harbor their own genetic material. Mitochondrial DNA (mtDNA) encodes a small number of polypeptides needed for the electron transfer chain (ETC). The ETC is responsible for cellular energy synthesis via oxidative phosphorylation (OXPHOS), during which some of the electrons leak from the ETC and are captured by oxygen to form reactive oxygen species (ROS) [1]. Most ROS are detoxified by cellular antioxidant defenses, but some escape and cause damage to cellular biomolecules like lipids, protein and nucleic acids [2]. Mitochondrial DNA may be particularly susceptible to such oxidative insult due to its proximity to the ROS production sites of the ETC [3]. Oxidative damage of mtDNA and its implications on cellular aging form the basis of the mitochondrial Free Radical Theory of Aging (mFRTA) [3]. One of the predictions of the mFRTA is the possibility of ROS ‘vicious cycle’ (Figure 1), referring to the hypothesized positive feedback mechanism in which mtDNA mutations cause an increase in the ROS production resulting in a higher *de novo* mutation rate [3]. Major challenges and questions with respect to the mFRTA have been summarized in some of the recent reviews [4],[5].

Despite uncertainties related to the assumptions of mFRTA, the importance of mitochondria as both the source and target of ROS in aging is supported by some transgenic mouse studies. For example, a 15% increase in the maximum and median lifespan is observed in knock-in mice expressing human catalase, an enzyme that decomposes H_2_O_2_ into water and oxygen, in mitochondria (MCAT), but not in the nucleus or the peroxisome [6]. Furthermore, MCAT mice heart tissue accumulates less than 50% of the mtDNA point mutations of age-matched wild-type mice [7]. Also, studies of homozygous knock-in mice with an error-prone polymerase-γ (POLG mutator mice) show that a dramatic increase in mtDNA mutation burden, most importantly deletions [7], is associated with shortened lifespan and some phenotypes that may resemble accelerated human-like aging [8],[9].

Although there is reasonable evidence for an age-dependent increase in mtDNA mutations, the dynamics by which these mutations accumulate is still largely unclear. Inferring dynamics and more importantly, the mechanism by which mtDNA mutations accumulate critically depends on accurate quantification of oxidative and mutational burden, which poses significant experimental challenges [4]. Many of these challenges stem from the limitations associated with experimental protocols in measuring oxidative damages and mutational frequency [10],[11], which typically exist at extremely low magnitude. Consequently, published reports show conflicting results regarding the levels of oxidative damages and mutation dynamics of mtDNA during aging [12]–[14]. A highly sensitive method based on the random mutation capture (RMC) assay has recently been developed for the quantification of mtDNA mutation frequency [15]. This method is based on restriction enzyme digestion and amplification of mtDNA molecules carrying mutations at the corresponding recognition site [12]. Application to wild-type mice has revealed mtDNA mutation burdens that were two orders of magnitude lower than previously determined using PCR-cloning and sequencing protocols [8],[9]. This indicates that PCR artifacts may have been a major contributor of errors in the past reports. Furthermore, quantification of age-dependent accumulation of point mutation burdens using the RMC assay in wild-type mice suggested an exponential increase, apparently supporting the existence of a ‘vicious cycle’ in the mutation accumulation [3],[13]. However, the low levels of burden suggest that point mutations may not be a major determinant of lifespan [12] and it is difficult to see how a positive feedback mechanism could set in at such a miniscule level of point mutation burden.

One requirement for addressing these uncertainties is a better understanding of the inherent stochasticity of cellular processes [16]. The accumulation of mtDNA mutations likely involves complex stochastic factors, such as the inherent random nature of mutations and related cellular processes in the context of aging. For instance, enzyme staining for ETC deficient tissue of substantia nigra neurons in aged subjects and Parkinson patients revealed a high degree of mosaicity of COX respiratory deficient cells [17]. This mosaicity has also been seen in skeletal muscle cells associated with sarcopenia in aged subjects [18]. Also, studies on *Caenorhabditis elegans* indicate that individual worms and their cells harbor a wide spectrum of mtDNA deletion loads [19].

Here we aim to address these challenges using a systems approach by way of constructing mathematical models that encompass the most relevant biological processes and also features related to experimental protocols to comprehend the origin and consequence of mutation variability that arises in individuals of a mouse population. Additionally, we seek to better understand the influence of intrinsic stochasticity of the mutation process on the variability observed in the experimental data. Such understanding may reveal possible causes of disagreements amongst published reports and further facilitate optimization of experimental design. In this study, we have constructed an *in silico* stochastic mouse model using the Chemical Master Equation (CME) [20]. Here, the accumulation of point mutations in mtDNA is simulated to arise as a consequence of what we believe to be a minimal process required for the maintenance of mtDNA integrity.

## Methods

### *In silico* mouse model

**Figure 2:**
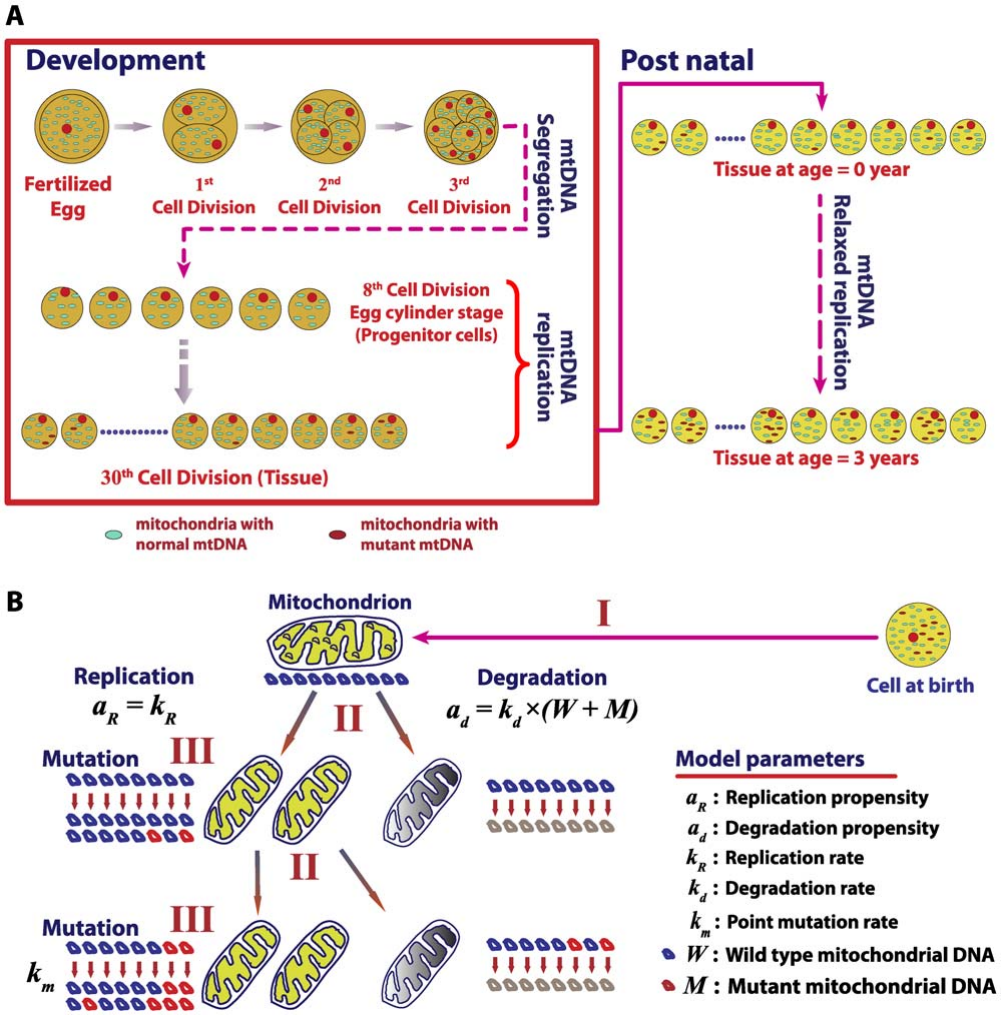
Stochastic mouse mtDNA model. (A) The *in silico* mouse model simulates the point mutation load of mtDNA in cells of a tissue such as heart and liver during development and postnatal stages. (B) Stochastic drift of point mutations in cells results as a consequence of mtDNA maintenance processes. Three sources of randomness are captured: (I) a random selection of a mitochondrion with ten mtDNA molecules from a well-mixed population, (II) a random replication or degradation of a mitochondrion, and (III) random occurrences of *de novo* mtDNA point mutation during replication.

The *in silico* mouse model accounts for the accumulation of mtDNA point mutations across two stages of mouse life: development and postnatal (Figure 2). In this study, the number of wild-type mtDNA (*W*) and mutant mtDNA (*M*) molecules are tracked for each cell in whole mouse heart (∼2.5×10^7^ cells) and liver tissues (∼4×10^8^ cells) [21]. Each mutant mtDNA molecule is assumed to contain only a single mutation in the *Taq*I recognition site (TCGA), following the RMC experimental design [12]. The probability of finding two or more mutations at the same site is negligible [15]. The model simulates two mtDNA-related maintenance processes: mitochondrial turnover, comprising of relaxed replication and degradation of mitochondria, and *de novo* point mutation, based on a minimal conservative assumptions. First, the mtDNA population of each cell is assumed to exist as a well-mixed pool due to fast fusion and fission dynamics of mitochondria [22]. Second, due to the low overall mutation burden, point mutation burden is assumed to remain below the level of functional significance (i.e. no nuclear retrograde signaling [23],[24]). While the latter assumption is conservative, our simulations indicate that the incorporation of functional effects of mutations into the model, by assuming that mutant mtDNA are non-functional and cells respond to a decrease in the number of wild-type (WT) mtDNA by increasing replication, does not result in any significant changes to the mutation burden (see Text S1 and Figure S3). A Langevin formulation using relaxed replication assumption demonstrated that stochastic drift can lead to a clonal expansion of mtDNA mutations in human [25].

Following experimental evidence, each mitochondrion is assumed to carry 10 mtDNA molecules and these mtDNA are assumed to undergo replication and degradation due to mitochondrial turnover [26]. In a turnover event (Figure 2B), ten molecules of mtDNA are chosen randomly from a well mixed population of mtDNA in a cell and are either degraded or replicated according to the CME described below. The selection of ten wild-type and mutant mtDNA molecules from the population can be described as a hypergeometric random sampling following the probability distribution: [27]

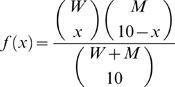
(1)where *x* represents the number of wild type mtDNA chosen for replication or degradation.

*De novo* point mutation can occur during replication of mtDNA due to mis-pairing associated with ROS-induced mutagenic lesions such as 8-hydroxy-2-deoxyguanosine (8OHdG) [2] or as random errors arising due to finite polymerase-γ (POLG) fidelity [28]. Consequently, each replication of a wild-type mtDNA has a finite probability, given by the mutation rate constant (*k_m_*), to produce a mutant. Here, the number of *de novo* mutant mtDNA is randomly chosen from a binomial distribution: [27]

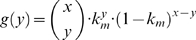
(2)where *y* denotes the number of *de novo* mutations resulting from replication of *x* wild-type mtDNA.

Based on these probabilities, the *in silico* mouse model is formulated as a CME in which each mtDNA-related process: replication without mutation, replication with *de novo* mutations and degradation, is described as a jump Markov process with the following state transitions: 
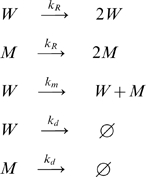
(3)The first two transitions reflect replication without mutation, the third represents *de novo* mutation, and the last pair represents degradation. A general formulation of CME is given by: [20]

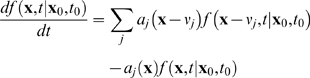
(4)where 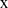 is the state vector denoting the total number of each molecular species present in the system and the function 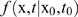 denotes the probability of a system to assume the state configuration 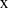 at time *t*, given the initial condition 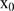 at time 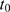. The function *a_j_* denotes the propensity function, while 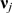 is the state change associated with a single *j*-th event. The propensity function 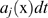 gives the probability of the *j*-th event to occur in the time interval [*t, t+dt*). As analytical solution to CME is usually not available even for moderately sized systems [29], Monte Carlo algorithms have been employed to solve the CME numerically [30], e.g. using Gillespie's SSA (Stochastic Simulation Algorithm) [31]. In SSA, two random variables (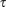, *j* ) determine the temporal evolution of the states in a system, where 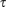 is the time for the next event to occur and *j* is the type of event that will take place. The probability density functions of 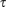 and *j* are evaluated based on the propensity function of the events involved [29].

A modified version of the SSA is used in this work for simulating *in silico* mice tissues based on the following CME:
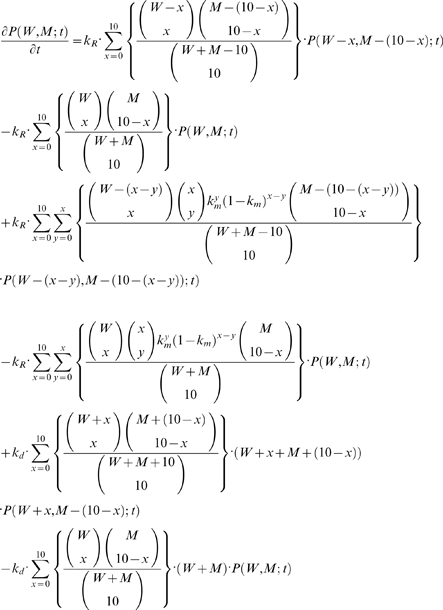
(5)


The density function 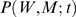 denotes the probability of a cell in a given tissue to contain *W* and *M* number of wild-type and mutant mtDNA, respectively, given the initial conditions of the states (not explicitly stated here for brevity, refer Equation 4). The parameters *k_R_*, *k_d_* and *k_m_* represent the specific probability rate constants for mtDNA replication, degradation and *de-novo* point mutations, respectively. The terms in the curly braces describe the hypergeometric sampling of mtDNA from the population. Particularly, the first two terms of the CME above represent mtDNA replication without mutation, the second pair of terms corresponds to replication with *de-novo* mutation, and the last two terms represent the degradation of mtDNA. The CME can be solved numerically using a Monte Carlo approach following the SSA. The implementation of the modified SSA is described below:

1. Compute the propensities of replication and degradation processes as a function of *W* and *M* at time *t*.
2. Based on the propensities, generate random samples of (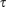, *j* ) as in the SSA algorithm [31].
3. Select ten mtDNA molecules randomly from the population (hypergeometric sampling) for either replication or degradation accordingly. Each replication of a wild type mtDNA can result in a mutant mtDNA with a probability given by the mutation rate constant (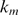).
4. Update *W* and *M* based on events in steps 2 and 3 and increment the time *t* by 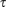.
5. Repeat steps 1 through 4 until the desired end time.

To predict mtDNA mutation burden in a single organ or tissue, millions of such simulations are performed to capture the mtDNA dynamics of all cells in a tissue.

Simulations were performed using an IBM high performance computing cluster with 112 Intel 1.6 GHz processors. The simulation code (Text S2) was compiled using GNU FORTRAN compiler G77 (v4.1.1) and run on a CentOS Linux platform. On average, a single simulation of a heart tissue (∼25 million cells) from development to 3 years of age required approximately 3 hours.

### Simulations of mouse development

The embryonic cell divisions begin after fertilization of an oocyte. Mouse oocytes harbor a large number of mitochondria (∼1.5×10^5^ mtDNA) [32], which allow the zygote to multiply initially without the need to replicate mtDNA [33],[34]. Mouse embryos with dysfunctional mitochondrial replication are able to proceed through the implantation and gastrulation stages, but eventually die, presumably when the mtDNA synthesis becomes necessary to maintain ATP level [35],[36]. Furthermore, the total mtDNA number in mouse embryo does not increase until the late stage of blastocyst, which is roughly the 7^th^ to 8^th^ cell divisions in development (i.e., 4.7 to 5.5 days post coitum (d.p.c)) [33],[34],[37]. During these stages, mtDNA are segregated among the dividing progenitor cells (Figure 2A). Consequently, each progenitor cell of the developing embryo has only few copies of mtDNA at the early egg-cylinder stage [33],[34].

In order to account for the mtDNA segregation without replication during the initial cell divisions, the developmental simulations start from the end of the 8^th^ stage (5 d.p.c) with an initial wild-type mtDNA count of roughly 580 molecules per cell (*W* = 580, *M* = 0) [33]. Mitochondrial DNA replication is tied to the cellular division to maintain a steady state number of total mtDNA after each division [38]. Mouse development lasts until 20 d.p.c [39] with a doubling time of roughly 15.5 hours [40]. The mtDNA replication rate is estimated assuming that mtDNA doubles its population every 15 hours while still undergoing degradation. Here, a cell division occurs when the total number of mtDNA count reaches twice the steady state homeostatic count (Table 1). The segregation of wild-type and mutant mtDNA between the daughter cells is assumed to occur at random, without any selective advantage according to a hypergeometric distribution: [27]

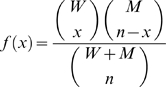
(6)where 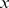 denotes the number of wild-type mtDNA in one of the daughter cells after segregation and *n* is the total number of mtDNA in a single daughter cell (i.e., *n = *(*W+M*)*/*2). During development, polymerase-γ, the care taker of the mtDNA replication fidelity, is the main contributor for point mutations in mtDNA, with negligible oxidative activity and damage [28],[41].

**Table 1:** Basic model parameters of the *in silico* stochastic mouse model.

Rates	Unit	Values	Comments	References
*W_0_*	*molecules*	580	Initial value of wild type mtDNA during start of development	[33],[64]
*M_0_*	*molecules*	0	Initial value of mutant mtDNA during start of development	
*k_d_*	*d^−1^*	2.3377×10^−3^	Degradation rate of mtDNA	[45]
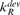	*molecules d^−1^*	465	Maximum replication rate of mtDNA during development	
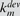	*rep^−1^ bp^−1^*	1.0×10^−7^	Mutation rate of mtDNA during development (POLG fidelity)	[28],[41],[43]
*N_cyc_*	-	22	Number of developmental cycles	[37],[39],[40],[64]
*(W+M)_ss_*	*molecules*	3500	Homeostatic set-point of the mtDNA population (Heart cells)	[21],[65]
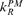	*molecules d^−1^*	0.8182	Maximum replication rate of mtDNA during post natal stage	
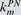	*rep^−1^ bp^−1^*	1.6×10^−6^	Mutation rate of mtDNA during post natal stage (POLG fidelity and Oxidative burden)	[11],[28],[41],[43],[54],[55]
*N_cell_*	-	2.2443×10^7^	Number of cells (Heart)	[21],[61]
α*_POLG_*	-	200	POLG allele fidelity factor	[28],[41],[43]

### Simulation of postnatal stage

After birth, many tissues like heart do not undergo further cellular division. However, mtDNA in these tissues are still continuously turned over independent of cellular division, a process called “relaxed replication” [26]. The functional significance of relaxed replication in postmitotic tissues like heart and brain is to maintain a healthy population of mtDNA to satisfy the cellular energy requirements [26],[42]. The postmitotic simulations continue from cells produced at the last stage of development (Figure 2A), in which each cell maintains mitochondrial biogenesis to balance degradation. The mutation rate in this stage is a summation of contributions from oxidative damage and POLG-related error.

### Simulation of POLG mutator mouse models

The *in silico* mouse model is also used to simulate POLG mutator heterozygous (POLG^+/mut^) and homozygous (POLG^mut/mut^) mice by changing the rate of *de novo* point mutations. Mutator mice carry a proofreading-deficient allele of POLG which has 200 times the error rate of the wild-type enzyme [28],[43]. Thus, in the simulations of POLG mutator mice, the model formulation remains the same in all aspects with the exception that the POLG error rate corresponding to the mutant allele is assumed to be 200 times higher (Table S2 and S3). In heterozygous POLG mutator mouse, the replication of mtDNA molecules is carried out by either wild-type or mutant allele with equal probability.

### Model parameters

Model parameters are compiled from published data for mice and we have ensured that they are consistent with the current literature and the state of the art techniques. The basic model parameters are listed in Table 1, while more detailed information of the rest of parameters used in all mouse models is given in Tables S1, S2 and S3.

#### Mitochondrial DNA degradation rate (*k_d_*)

Cellular organelles like mitochondria are normally degraded by the autophagy process, where an entire organelle is engulfed by a lysosome and undergone lytic degradation [44]. The half-life of mouse mtDNA molecules can be studied *in vivo* using isotopic deuterated water ^2^H_2_0 [45]. The decrease of isotopic deoxyadenosine in mtDNA after discontinuation of ^2^H_2_0 treatment can be used to determine the turnover of mtDNA [45],[46], providing a highly sensitive measurement of mtDNA degradation rate constant for the model, *k_d_*.

Hepatocytes of liver are mitotically quiescent and stop differentiating at the end of the postnatal growth period (∼60 days in the rats) [47],[48]. While under normal conditions these cells have a very long life span (∼400 days) [48], they can become mitotic in response to hepatic stress or injury [47],[48]. Thus, in simulating the liver tissue, the slow cellular turnover is approximated using an elevated mtDNA turnover (Table S2).

Literature values regarding mitochondrial turnover differ widely, citing half life values ranging from 6 days to ∼300 days [45], [46], [49]–[51]. The literature is relatively sparse and spans many decades. Consequently the methods utilized and the tissues examined differ significantly among studies, making direct comparison somewhat difficult. However, considering the higher value of turnover [49],[50], a simple estimation of the expected mutation load under the simplifying assumptions (refer to Text S1 for details), show that unless there is a preferential degradation of mutants against the wild-type mtDNA (which at least can be envisioned for the functionally relevant mtDNA mutations), it is difficult to see how such high turnover rates can be physiologically feasible given the low mtDNA mutation frequencies that are actually found by RMC assay [12] (Figure S6). We also intend to explore such mechanisms, which are likely to be dependent on the compartmentalization effects of mitochondria (fusion-fission dynamics), retrograde signaling [24] and mitochondrial threshold levels [52].

#### Mitochondrial DNA replication rate (*k_R_*)

The mtDNA copy number is maintained throughout the cell growth and divisions [53]. The mtDNA replication should occur to balance the degradation. There exist evidence supporting the existence of a retrograde signaling between mitochondria and nucleus to regulate the mtDNA content based on cellular bioenergetics [24]. This suggests that mitochondrial biogenesis may be initiated as soon as the mtDNA copy numbers in a cell falls below a certain homeostatic set-point value. Here, we have used a constant biogenesis (i.e. without retrograde signaling), but the main conclusions of our work remain the same even with retrograde signaling (see Figure S3 and Text S1). The constant mtDNA replication rate was deduced based on the homeostatic mtDNA copy number in a cell and the degradation rate of mtDNA. Thus, the replication constant *k_R_* is given by:
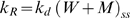
(7)where 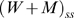 represents the homeostatic level of mtDNA population in the cell (Table 1).

#### Mitochondrial DNA point mutation rate (*k_m_*)

*In vivo*, 8OHdG level most likely ranges from 0.3 to 4.2 lesions per 10^6^ bases in nuclear DNA [11],[54],[55]. However, such lesions make only about 10 to 20% of the complete damage spectra [12],[56]. Therefore, the actual frequency of point mutation rate may be as low as 1.5 and as high as 42 lesions per 10^6^ DNA bases per replication. In this work, we have made a conservative assumption that the oxidative damage to mtDNA is the same as that to nuclear DNA, consistent with our earlier observations [57]. While some reported values of 8OHdG adducts in mtDNA that are an order of magnitude higher than in nuclear DNA [10], our simulations indicate that such a high damage level is unlikely as this will lead to an mtDNA mutation burden much in excess of those quantified by RMC assay (Figure S5) [12].

In addition to the oxidative damage, the fidelity of polymerase-γ also contributes to *de novo* point mutations during replication. The polymerase is responsible for the replication and proof reading of newly synthesized strands with a reported error rate between 1×10^−7^ and 1×10^−6^ bp^−1^replication^−1^ for the wild-type enzyme [28]. Therefore, the overall mutation rate is a sum of oxidative damage and POLG-related errors, giving a range of mutation rate between 1.6×10^−6^ and 4.3×10^−5^ mutations per base pair per mtDNA replication. A conservative value (lowest) of 1.6(10-6 bp-1 replication-1 is chosen for wild-type mouse simulations.

The summary of all the parameters used in this work is described in Table S1, S2 and S3. Table S1 gives the details on the model parameters used for wild-type mouse simulations. Table S2 and S3 give a summary of the model parameters used for simulating the POLG mutator mice, POLG heterozygous (POLG^+/mut^) and POLG Homozygous (POLG^mut/mut^), respectively.

## Results/Discussion

### Statistical features of the RMC assay

**Figure 3:**
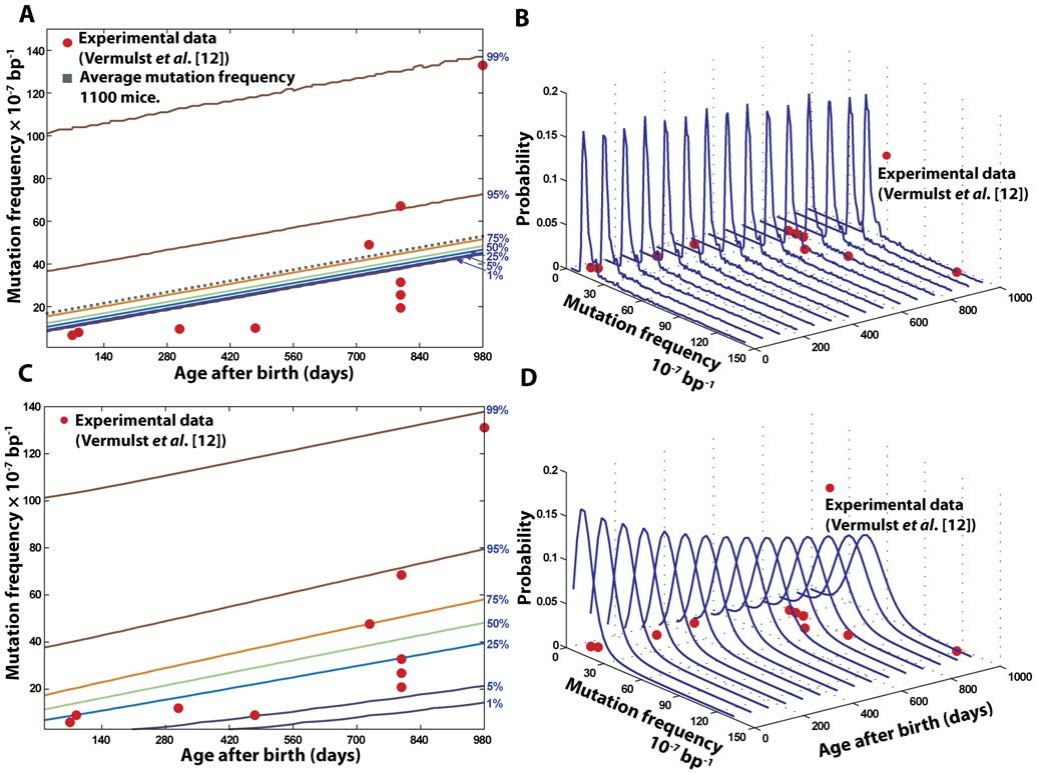
Stochastic determinants of age-dependent dynamics in the observed mtDNA point mutation frequency. Heart tissue simulations provided the distribution of mutation frequency among 1,100 *in-silico* wild type mice. (A, B) The percentiles and probability distribution functions of the mutation frequency arising from the intrinsic stochasticity of cellular processes alone. The dotted line indicates the evolution of the average mutation frequency of 1,100 mice, which grows linearly with time. (C, D) The percentiles and probability distribution functions of the mutation frequency in the RMC assay of *in silico* wild-type mouse population. The apparent variability arises from the combined effect of intrinsic stochasticity and the (hypergeometric) sampling variability in the RMC protocol (details in the main text).

*In silico* wild-type (WT) mouse population of 1100 individuals was generated starting from embryo up to three years of age, the approximate life span of mice (Figure 2). The overall point mutation frequency in 2.5×10^7^ cells of whole heart tissues was recorded at the end of each cell division during development and every fortnight during the postnatal stage. Figure 3 illustrates the percentile and distribution function of the mutation frequency arising from two important sources of variability related to the quantification of mtDNA point mutations. The probability density functions indicate the distribution of mutation frequencies in the population as a function of time. Each contour on the percentile plot represents the maximum mutation frequency that a given percentage of the population harbors (e.g. 99% of mice harbor mutation frequencies up to and including the level indicated by the 99^th^ percentile curve (Figure 3A, 3C)).

**Figure 4:**
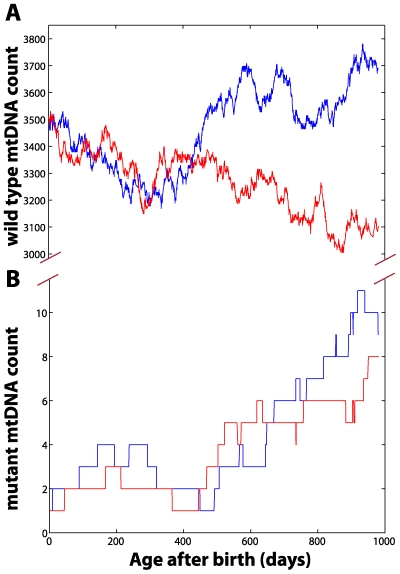
Stochastic evolution of mtDNA states. (A) represents the stochastic evolution of the wild-type mtDNA, while (B) illustrates the stochastic changes in the mutant mtDNA population. Red and blue curves indicate the outcomes of two independent realizations.

The main source of randomness is the intrinsic stochastic nature of the aging process, which arises from the mtDNA maintenance processes (Figure 2B). Note that the intrinsic stochasticity prevailing in the *in silico* population has a long tailed non-Gaussian density function (Figure 3B, 3D), indicating that a small fraction of the population harbors a significantly higher mutation burden. Cell-to-cell variability of mtDNA mutation load is also observed as a result of the random processes (Figure S1). Figure 4 illustrates the evolution of mtDNA states (*W* and *M*) in two cardiomyocytes during the postnatal stage of a mouse. Random fluctuation of wild-type mtDNA can be seen in the population with regular bursts and decay of mutant mtDNA. Furthermore, it is interesting to observe that despite the significant cell-to-cell variability of mutation load being large (Figure S1), the average accumulation of mtDNA mutation in tissue remains linear after birth (Figure 3A). Also, the variance due to the natural aging process remains roughly constant during the mouse life span, indicating that the variability among individuals is inherited at birth.

However, for comparison with data derived from RMC assay, a second source of variability has to be considered due to the intrinsic statistical properties of the assay protocol. This is because, the determination of point mutation burden by the RMC assay involves drawing a random sample of mtDNA copies (∼840,000) from tissue homogenates [12]. This sampling procedure introduces additional variability that becomes significant due to the low overall count of total mtDNA mutations. This statistical feature of the RMC protocol can be described as sampling from a hypergeometric distribution [27]. 
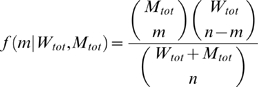
(8)where *m* denotes the number of mutant mtDNA molecules present in a random sample of mtDNA of size *n* (*n* = 840,000 mtDNA molecules in this case). Thus, for low mutation frequencies and sample sizes, the RMC protocol introduces significant additional variability in the data. For example, in heart tissue homogenate containing 10^10^ molecules of mtDNA with a mutation frequency of 10^−6^/bp (a total of 4×10^5^ mutant mtDNA), samples of 840,000 mtDNA drawn from the same homogenate will have a mean value of 3.36 mutants with a standard deviation of 1.83 molecules or 54.6% coefficient of variation from the RMC sampling alone.

The compounded effect of the two sources of variabilities (intrinsic aging related and RMC assay) can be expressed by,
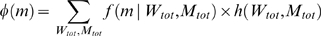
(9)where 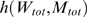 denotes the underlying probability distribution of mtDNA mutations predicted by the mouse model simulations and 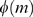 is the overall probability function of *measured* mtDNA mutations. Importantly, the additional variability associated with the sampling of mtDNA in the RMC protocol causes the mutation frequency variance to increase as a function of the average mutation frequency (Figure 3C), a result expected from a hypergeometric distribution. This is particularly relevant here because of the age-dependent increase in mean mutation burden and the fact that the distribution describing the mutation process is long-tailed (Figure 3A, 3B). When this underlying mutation dynamics is sampled using the RMC assay, the resulting data will exhibit an age-dependent increase in variance. Due to low number of replicates (typically *n*<5 per age group), it is highly probable to obtain data that are best approximated by a non-linear, possibly exponential model (Figure 3C). However, this apparent exponential increase is not actually a feature of the underlying mutation dynamics, which may be in fact, linear (Figure 3A). This has important implications for the interpretation of the available experimental data.

In accordance with the interpretation reached in the original experimental work [12], the variance in the *in silico* data as well as the experimental data for low *n*-values appears to suggest an exponential dynamics supporting the ‘vicious cycle’ theory [3],[13]. However, on careful consideration (Figure 3), the apparent exponential increase of the mutational burden is actually an artifact of: (a) intrinsic stochasticity of aging process (Figure 3A, 3B), coupled with (b) the random sampling variability introduced by the statistical properties of the RMC protocol (Figure 3C, 3D). Experimentally, it is not possible to carry out 100 s or 1000 s of repeats and it is therefore difficult to distinguish between a truly exponential and a linear increase of age dependent point mutation burden. In summary, while the RMC assay is able to quantify extremely low levels of mutations, its discrete nature (in terms of mutant mtDNA count) introduces significant challenges in data analysis and interpretation. The interpretation of the data can be flawed if the statistical properties of the RMC assay are not considered. Taking both processes into consideration, the fundamental mtDNA maintenance processes modeled by our *in silico* mice are in excellent agreement with the published data (Figure 3C). However, the last data point of mutation burden from an old mouse (980 days) deviated from *in silico mouse* population (*p*-value = 0.064), suggesting that other processes not predicted by our model may be involved during the last months of life (e.g., inflammation or other disorders that can accelerate oxidative DNA damage [58]).

### Transgenic mouse studies

Transgenic mouse studies on POLG mutator mouse have recently shed some light on the role of mtDNA in aging [8],[9],[12]. However with these mutator models, many open questions still remain about the role of mtDNA mutation in aging. For example, only the homozygous mutator mice exhibited accelerated human-aging-like phenotypes (e.g., anemia, alopecia, kyphosis) and shortened lifespan, while the heterozygous mice have no obvious aging phenotypes, despite significantly elevated mutation burdens [9].

**Figure 5:**
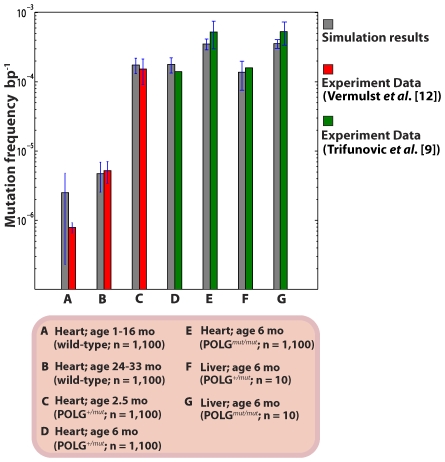
Average mtDNA point mutation frequencies in WT and POLG mutator mice. The variances in the *in silico* mouse data represent the intrinsic stochasticity only (without the RMC sampling variability).

After successfully validating the *in silico* mouse model against wild-type mouse data, we further simulated 1,100 hetero- and homozygous POLG mouse heart and liver tissues by elevating the baseline POLG error rate to 200 times that of wild-type [28],[43]. We found an excellent agreement of our *in silico* results with the reported mutation burdens from two different laboratories [9],[12] (Figure 5 and Figure S4). As with the wild-type mice, the point mutation increase was linear with age (Figure S4). Again, mitochondrial turnover and *de novo* point mutations alone were sufficient to explain the accumulation of mtDNA point mutations. These results indicate that even at the elevated levels of point mutations ROS-mediated acceleration of point mutations with age is not necessary to explain the data presented in [8],[9]. This is consistent with additional experimental observation suggesting that the levels of ROS in POLG mice are not significantly elevated in the mutator mice [8]. Crucially, no modification of mtDNA maintenance rate constants was required to reproduce the experimental data [8],[9]. That is, one does not have to resort to assumptions such as the existence of a vicious cycle or other possible feedback mechanism [59],[60].

### Significance of development

The stage in an organism's life from which the accumulation of mtDNA mutations starts to become functionally significant (if at all) is unclear. During development, mtDNA replication is tied to the cellular division, and as a consequence, initial mutations may arise as soon as mtDNA replication begins. In fact, the total number of replications during development is comparable to that during the entire adult life. In mice, the heart tissue develops in about 20 days [39]. Considering the degradation rate described in Table 1 and the mouse heart to contain ∼2.5×10^7^ cardiomyocytes [21],[61] arising from 22 cell divisions (6 progenitor cells), the total number of mtDNA replications needed to maintain homeostatic value of mtDNA (Table 1) [21] per cell should exceed 9×10^10^ times during the development. On the other hand, based on the degradation rate of mtDNA in postnatal stages (Table 1) [45], the number of mtDNA replications events over the three years lifespan of mice is about 1.3×10^11^. Thus depending on their source (ROS, POLG errors), the development period may carry comparable contributions in *de novo* mtDNA mutations as does the entire adult life.

**Figure 6:**
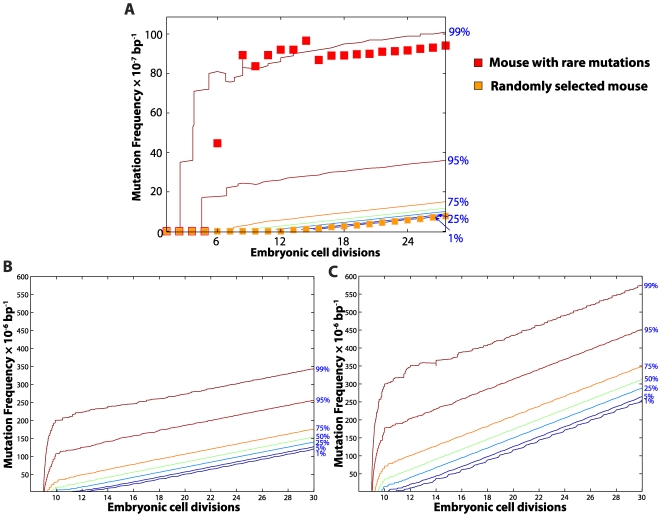
Mitochondrial DNA point mutation during mouse development. Expansion of mtDNA point mutations during heart tissue development from *in silico* wild-type (A), POLG^+/mut^ (B) and POLG^mut/mut^ mice (C) population (n = 1,100). (A) The square symbols show examples of point mutation trajectory from two different mice, one of which suffers from a rare point mutation early in the development, resulting in the amplification of the mutation frequency in subsequent cell divisions. (B) Like in the wild-type cohort, *de-novo* point mutations generated in the POLG^+/mut^ mice during the early cell divisions can accumulate very quickly, resulting in a high mutation load in the cells at birth. (C) Since the error rate of mtDNA replication in POLG^mut/mut^ is much higher than the wild-type mtDNA replication, a significant proportion of the population (>75%) harbors mtDNA mutations at an early stage of development (before the 10^th^ cell division). As a consequence, the resulting mutation load in the tissue is significantly higher than that in the wild-type tissues at birth.

POLG errors have been postulated to be the main cause of *de novo* point mutations in murine embryonic fibroblast [28],[41]. Therefore, the POLG baseline error rate was used as mutation rate during development. Generally, our *in silico* mouse data highlight that mutations occurring in the early embryonic cells have a strong impact on the mutation load at birth (Figure 6) and that the variability among individuals is set during development (Figure S2 and Figure S4). Since the mtDNA replication is several folds higher than the degradation during development, *de-novo* point mutations generated during the early cell divisions can accumulate very quickly, resulting in a high mutation load at birth in some individuals (Figure 6). These results highlight that the stochastic drift of mutation dynamics during the early developmental cell divisions may be a deciding factor of the organism's mutation trajectory, and also a major contributor of the mutation variability in a population, including isogenetic individuals [19]. The variability generated during development is conserved throughout the organism's life (see Figure 2A and Figure S4A, S4B).

**Figure 7:**
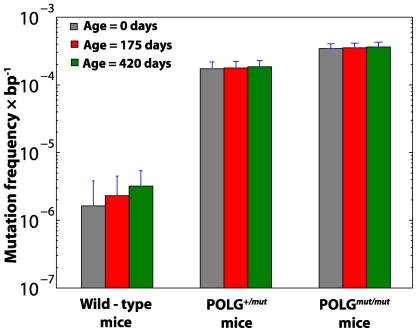
Average mtDNA point mutation accumulation in wild type and POLG mutator mouse models. Wild-type mice have a low mutation burden at birth, but they accumulate relatively more mutations during their life. On the other hand, the POLG mice harbor a significant mutation load at birth due to error-prone mtDNA replications during development. In the post-mitotic stage however, the relative accumulation (in comparison to mutations at the birth) is significantly lower due to the slow turnover of mtDNA.

In postmitotic tissues, like heart, mtDNA are continuously turned over independent of cellular division [26]. Although the turnover rate of mtDNA is lower during the postnatal stage than during development, the higher mutation rate due to oxidative damage (Table 1) can lead to 2–3 fold increase in the mutation load between birth and old age in wild-type mice (see Figure 2A and Figure 7). The *in silico* POLG mice however differ from the wild-type because in these mice, the POLG error is the dominant contributor of *de novo* point mutations, both during embryonic and postmitotic stages (Supplementary Table S2 and S3). Due to faster mtDNA replication (tied to cell division), most of the mutations in mutator mice therefore arise during development (Figure 6B, 6C and Figure 7). This is consistent with the experimental data which shows clearly that mutator mice are born with significantly elevated mutation burden [9],[62]. However, during their adult life, the accumulation is relatively lower compared to their development, due to the slow turnover of mtDNA [45].

Furthermore, the above observation leads to an interesting insight, largely unappreciated in the original work [8],[9],[63], regarding the point mutation load in tissues that remain mitotic (epidermal, stem cells, spleen). Since in POLG mice the point mutation burden of mtDNA is dominated by POLG errors, mutation accumulation in fast dividing cells is expected to be several fold faster than in postmitotic tissues such as heart. This is consistent with the experimental observation in POLG mutator mice, where some of the most prominent pathologies associated with the fast dividing tissues manifest in the form of alopecia, spleen enlargement and anemia. However it should be appreciated that such mechanistic hypothesis is speculative, because we have not included the simulation of mtDNA turnover of any fast dividing tissues in the present work. Treatment of cell division and selection pressure for mitochondrial turnover might be a promising area of investigation for the future work.

### Conclusions

By thinking carefully about the different sources of stochasticity in each process from early development all the way to experimental sampling, we have identified the RMC assay procedure as a major contributor to the overall uncertainty. In contrast to the original interpretation of the data, our analysis reveals that the existence of an exponential dynamics in point mutations cannot be inferred with certainty, and thus no contradiction between the observed point mutation dynamics and the apparent absence of evidence for elevated oxidative stress exists. A detailed, quantitative understanding of the relevant sources of noise also allows optimization of experimental designs, thereby opening avenues for maximizing information return and minimizing cost, time and animal use.

The fact that the reproduction of the POLG mouse data requires no modifications to the wild type model, other than the obvious decrease of the polymerase fidelity, suggests that elevation of the point mutation burden does not trigger fundamentally new processes. In particular, neither mutant replicative advantage nor the elevation of the ROS dynamics resulting from the increase of the point mutation burden is required to explain the POLG data. This is consistent with our current view on the mFRTA [4], showing little evidence for the existence of vicious cycle mechanism. Two further observations related to the POLG mice that have originally been seen as somewhat surprising, can also be explained. The first is the observation that dividing tissues seem to be more severely affected in POLG mice than postmitotic tissues [9],[63]. The second is the fact that most mtDNA mutations in the POLG mice are already present at birth with comparatively little further accumulation during adult life, when compared to its development [9],[62]. Quantitative analysis shows both of these observations to be consequences of the low turnover of mtDNA in postmitotic tissues of adult mice.

Finally, our *in silico* analysis reveals the importance of early development in determining the trajectory of mtDNA mutation burden. This is in sharp contrast to the common assumption that health and diseases are determined predominantly by the genome interacting with the environment. Here, we have demonstrated that *in silico* modeling can contribute significantly to analysis and understanding of experimental data as well as potentially help to design more effective methodology. We believe that this approach of “Computer Aided Thought” can contribute towards a fundamentally improved understanding of intrinsically challenging biological problems such as aging.

## Supporting Information

Figure S1 Point mutation distribution in cells of heart tissue from different *in silico* mice. Stacked distribution plots of the normal and mutant mtDNA counts (W,M) in an *in silico* mouse heart. Each plot represents the simulation outcome of a single heart tissue. Subplots on the left pane represent the complete distribution of all states in the cells and those on the right pane illustrate the states distribution for all the cells having at least one mutant mtDNA (M>0) (Note, the frequency of the cells having mutant mtDNA in a wild-type tissue is two orders of magnitude lower than the cells from the tissue of POLG mutator mice). Dispersion of mutation load in the cells has an increasing trend amongst the three different mouse models with the POLG^mut/mut^ mouse having the highest dispersion of mutant mtDNA states in the cells. The stochastic nature of mtDNA turnover has a significant contribution in the mutation load dispersion. (A, B) Distribution of mtDNA states in the tissue of wild-type mouse. (C, D) Distribution of the mtDNA states in the tissue of POLG^+/mut^ (heterozygous) mouse. (E, F) Distribution of the mtDNA states in the tissue of POLG^mut/mut^ (homozygous) mouse.
(1.84 MB TIF)

Figure S2 Simulations with exclusion of developmental phase. Sources of variability in the observed mtDNA point mutation frequency of 1,100 *in silico* wild-type mouse heart tissues without developmental *de novo* point mutations. The mutation frequencies were recorded every fortnight up to 36 months. (A) The percentile curve of mutation frequency due to the intrinsic stochasticity of aging process in the mouse population. The mtDNA maintenance during life did not cause any observable variability among the mouse population, as indicated by the overlapping percentile curves and the sharp distribution. This is in agreement with our previous observation that the genetic variability is inherited from the development and conserved during the adult life. (B) The percentile curve of the mutation frequency in the RMC assay of *in silico* wild-type mouse population. The variability again increases with age due to the increase of the mutation frequency. The model excluding the development was in worse agreement with the experimental data than the trials that included the development (Figure 3 in Main Text). (C) Comparison of the average mutation frequency in mouse heart tissues with and without developmental contribution. Two types of simulations were performed, one type included *de novo* mutations during development while the other did not (i.e. no mutations at birth). Although the influence of development in the wild-type mice is rather insignificant at older ages, the exclusion of the developmental stage in the simulations of the POLG mutator mice causes a significant difference in the resulting mutation burden. The variance in the *in silico* mice data represents the intrinsic genetic variability only, without the RMC sampling variability.
(1.14 MB TIF)

Figure S3 Effect of different choices of point mutation model on the average mutation burden. The average mutation frequency reported in the plot represents the mutation burden in the heart tissues of wild-type mice and was recorded at the end of 36 months. (a) Comparison of the average mutation burden obtained using two different turnover rates. The higher turnover rate was based on a frequently cited experimental data (a half-life of 17 days reported by [1],[2] in the figure). It is evident that usage of such high value of turnover can give vastly differing mutation loads and the model is particularly very sensitive with respect to the turnover rates. Also Supplementary Figure S6 further illustrates the variation of the point mutation burden in population of 100 mice for this turnover rate. (b) Comparison of the average mutation burden obtained using two different replication models described in the supplementary text. The two replication models based on: (i) constant biogenesis, and (ii) biogenesis with the Hill-type kinetics are equivalent.
(1.29 MB TIF)

Figure S4 Stochastic determinants of age-dependent point mutation dynamics in mutator mice. Comparison of the observed mtDNA point mutation frequency in a population of 1,100 *in silico* POLG mouse heart tissues (Heterozygous and Homozygous). The mutation frequencies were recorded every fortnight up to 36 months (heterozygous) and 14 months (homozygous). (A) The percentile curves of the mutation frequency in the RMC assay of *in silico* POLG^+/mut^ mouse (heterozygous) population. The apparent variability arises from the genetic variations intrinsic to the aging process and the hypergeometric sampling variability in the RMC protocol (details in the Methods section). (B) The percentile curves of the mutation frequency in the RMC assay of *in silico* POLG^mut/mut^ mouse (homozygous) population. Unlike the wild-type, the uncertainty arising due to the combined effect of the two sources of variability does not increase with time. The variance remains roughly constant with age and this is primarily due to the high point mutation load prevailing in the cells at birth, which only increases relatively marginally with age.
(0.50 MB TIF)

Figure S5 Mitochondrial DNA point mutation burden under an elevated oxidative burden assumption. The oxidative burden used in these simulations was elevated to 10 times higher than that used in the main article (Figure 3 in Main Text). The mutation frequencies were again recorded every fortnight up to 36 months. (A, B) The percentile curve and distribution of the mutation frequency due to the intrinsic stochasticity of aging process in the mouse population (n = 500 mice). (C, D) The percentile curve and distribution of the mutation frequency estimated by the RMC assay of the *in silico* wild-type mouse population considered.
(1.12 MB TIF)

Figure S6 Mitochondrial DNA point mutation burden simulated using mtDNA half-life of 17 days. Inherent mtDNA point mutation frequency in the heart tissues of 100 mice using an mtDNA half life of 17 days (based on the references [2] and [3] indicated in the figure). The mutation frequencies in the post development were monitored every fortnight up to 36 months. (A) Expansion of the mtDNA point mutations during the heart tissue development and, (B) Point mutation dynamics in the mouse population post-birth.
(1.23 MB TIF)

Table S1 Model parameters used in the simulations of the *in silico* wild-type mice.
(0.07 MB DOC)

Table S2 Model parameters used in the simulations of the *in silico* POLG heterozygous mice's (POLG^+/mut^) mice.
(0.09 MB DOC)

Table S3 Model parameters used in the simulations of the *in silico* POLG homozygous mice's (POLG^mut/mut^) mice.
(0.07 MB DOC)

Text S1 Supporting Information
(0.07 MB DOC)

Text S2 Stochastic mtDNA Point Mutation Simulation Algorithm
(0.03 MB PDF)
