# Supplementary figures and images for: Stochastic Drift in Mitochondrial DNA Point Mutations: A Novel Perspective Ex Silico
Source: PLoS Comput Biol. 2009 Nov 20;5(11):e1000572. doi: 10.1371/journal.pcbi.1000572 (PMC2771766; doi:10.1371/journal.pcbi.1000572)

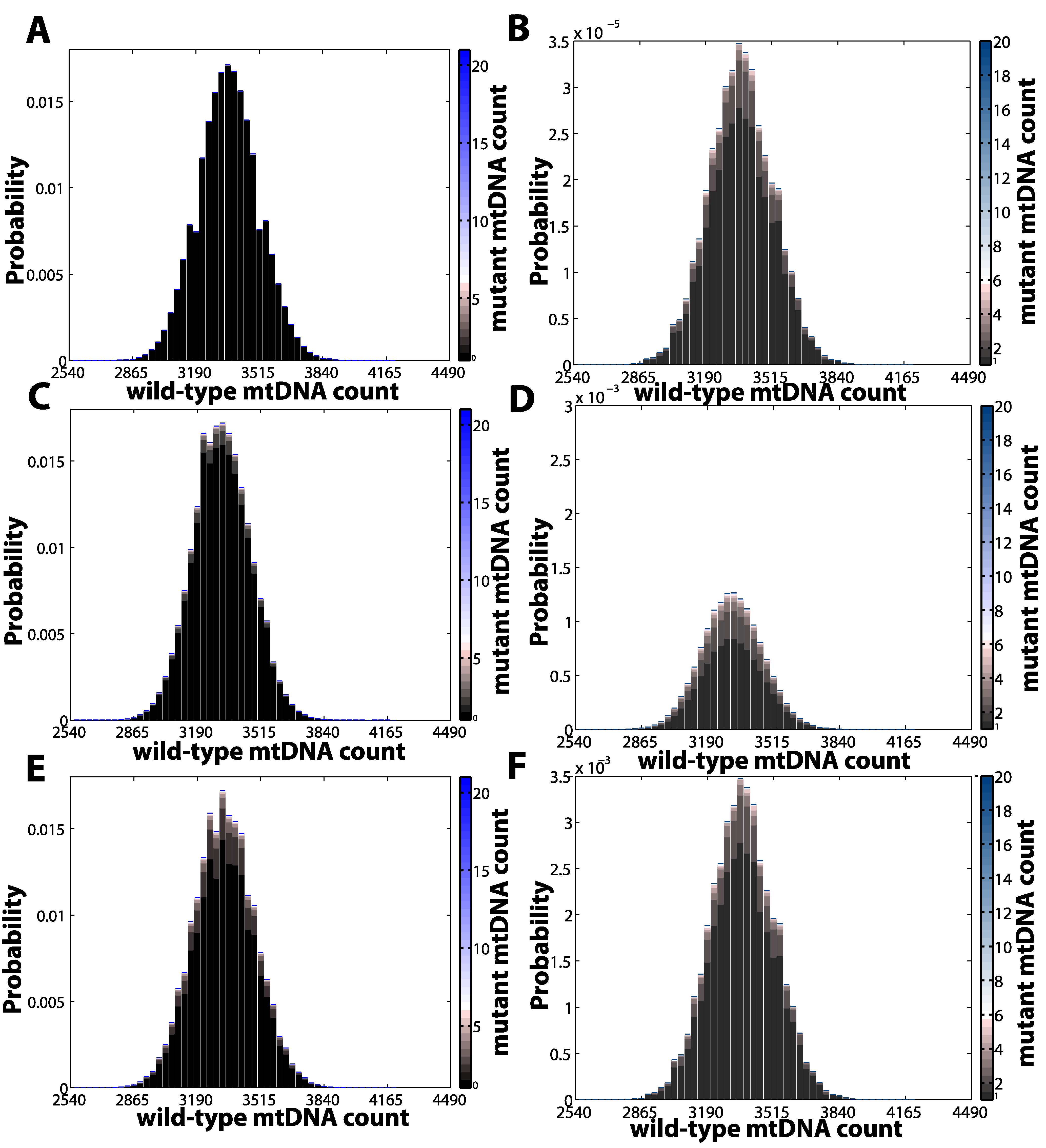

Supplement: Figure S1 — Point mutation distribution in cells of heart tissue from different in silico mice. Stacked distribution plots of the normal and mutant mtDNA counts (W,M) in an in silico mouse heart. Each plot represents the simulation outcome of a single heart tissue. Subplots on the left pane represent the complete distribution of all states in the cells and those on the right pane illustrate the states distribution for all the cells having at least one mutant mtDNA (M>0) (Note, the frequency of the cells having mutant mtDNA in a wild-type tissue is two orders of magnitude lower than the cells from the tissue of POLG mutator mice). Dispersion of mutation load in the cells has an increasing trend amongst the three different mouse models with the POLGmut/mut mouse having the highest dispersion of mutant mtDNA states in the cells. The stochastic nature of mtDNA turnover has a significant contribution in the mutation load dispersion. (A, B) Distribution of mtDNA states in the tissue of wild-type mouse. (C, D) Distribution of the mtDNA states in the tissue of POLG+/mut (heterozygous) mouse. (E, F) Distribution of the mtDNA states in the tissue of POLGmut/mut (homozygous) mouse. (1.84 MB TIF) [file pcbi.1000572.s001.tif]

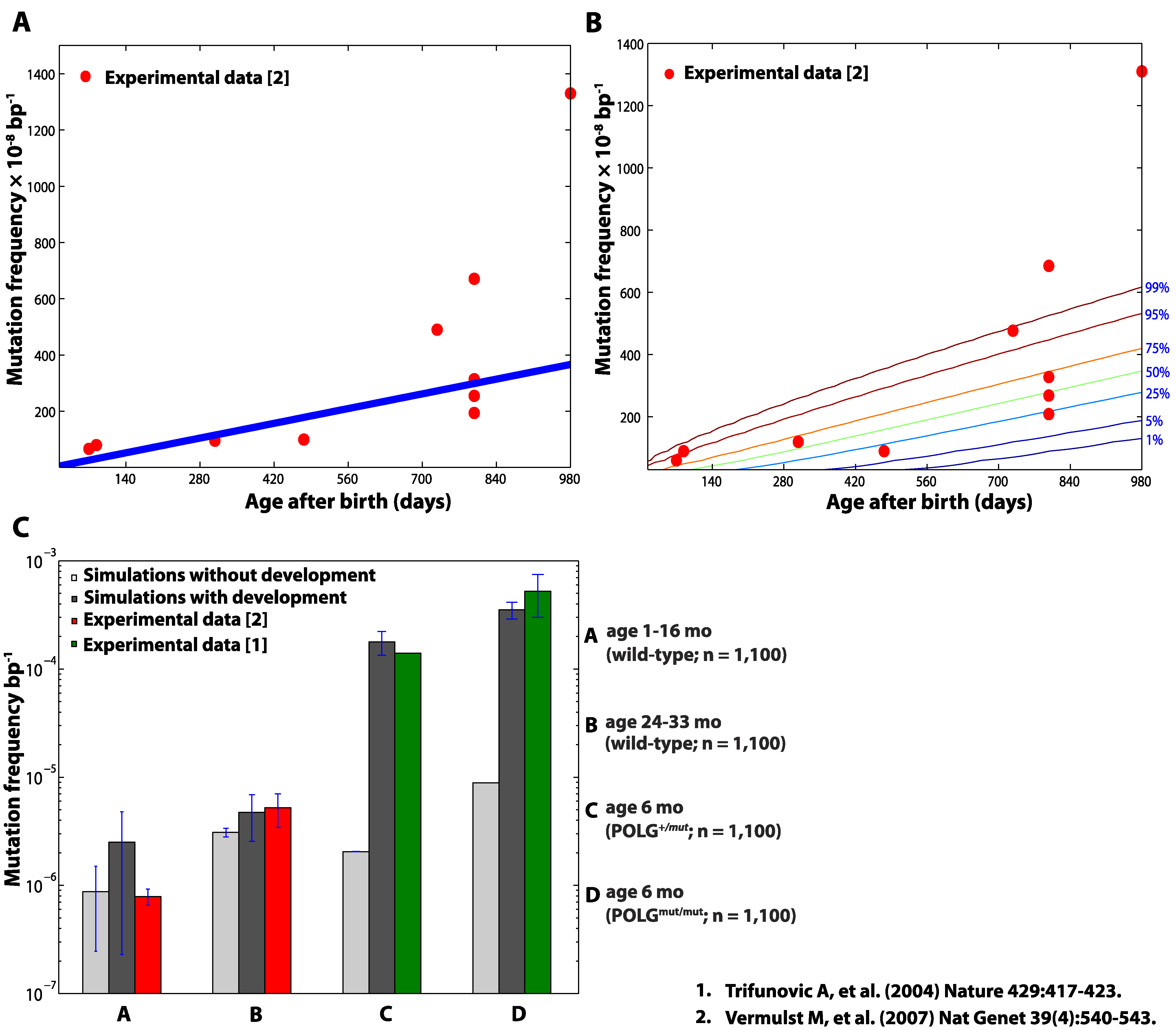

Supplement: Figure S2 — Simulations with exclusion of developmental phase. Sources of variability in the observed mtDNA point mutation frequency of 1,100 in silico wild-type mouse heart tissues without developmental de novo point mutations. The mutation frequencies were recorded every fortnight up to 36 months. (A) The percentile curve of mutation frequency due to the intrinsic stochasticity of aging process in the mouse population. The mtDNA maintenance during life did not cause any observable variability among the mouse population, as indicated by the overlapping percentile curves and the sharp distribution. This is in agreement with our previous observation that the genetic variability is inherited from the development and conserved during the adult life. (B) The percentile curve of the mutation frequency in the RMC assay of in silico wild-type mouse population. The variability again increases with age due to the increase of the mutation frequency. The model excluding the development was in worse agreement with the experimental data than the trials that included the development (Figure 3 in Main Text). (C) Comparison of the average mutation frequency in mouse heart tissues with and without developmental contribution. Two types of simulations were performed, one type included de novo mutations during development while the other did not (i.e. no mutations at birth). Although the influence of development in the wild-type mice is rather insignificant at older ages, the exclusion of the developmental stage in the simulations of the POLG mutator mice causes a significant difference in the resulting mutation burden. The variance in the in silico mice data represents the intrinsic genetic variability only, without the RMC sampling variability. (1.14 MB TIF) [file pcbi.1000572.s002.tif]

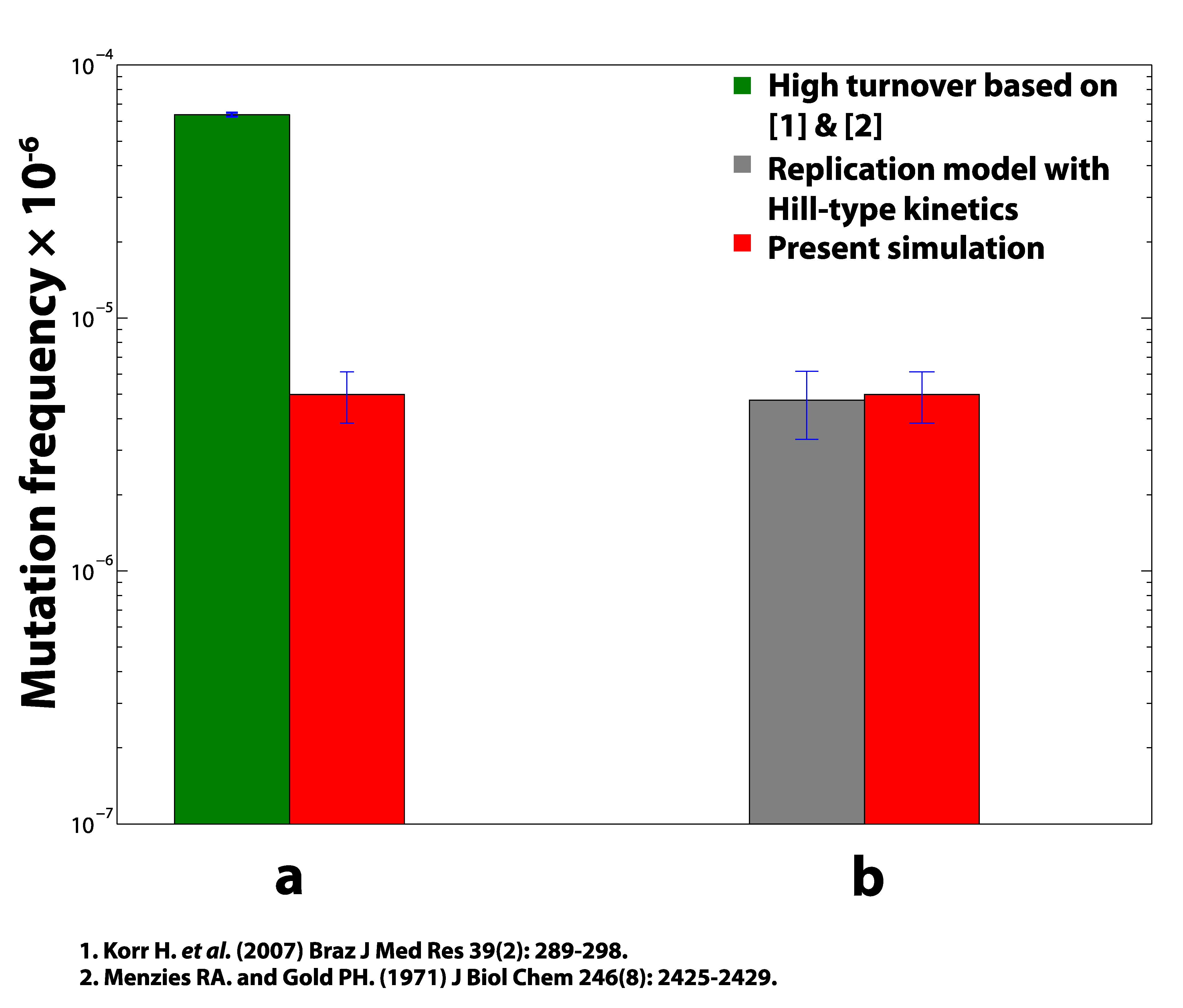

Supplement: Figure S3 — Effect of different choices of point mutation model on the average mutation burden. The average mutation frequency reported in the plot represents the mutation burden in the heart tissues of wild-type mice and was recorded at the end of 36 months. (a) Comparison of the average mutation burden obtained using two different turnover rates. The higher turnover rate was based on a frequently cited experimental data (a half-life of 17 days reported by [1],[2] in the figure). It is evident that usage of such high value of turnover can give vastly differing mutation loads and the model is particularly very sensitive with respect to the turnover rates. Also Supplementary Figure S6 further illustrates the variation of the point mutation burden in population of 100 mice for this turnover rate. (b) Comparison of the average mutation burden obtained using two different replication models described in the supplementary text. The two replication models based on: (i) constant biogenesis, and (ii) biogenesis with the Hill-type kinetics are equivalent. (1.29 MB TIF) [file pcbi.1000572.s003.tif]

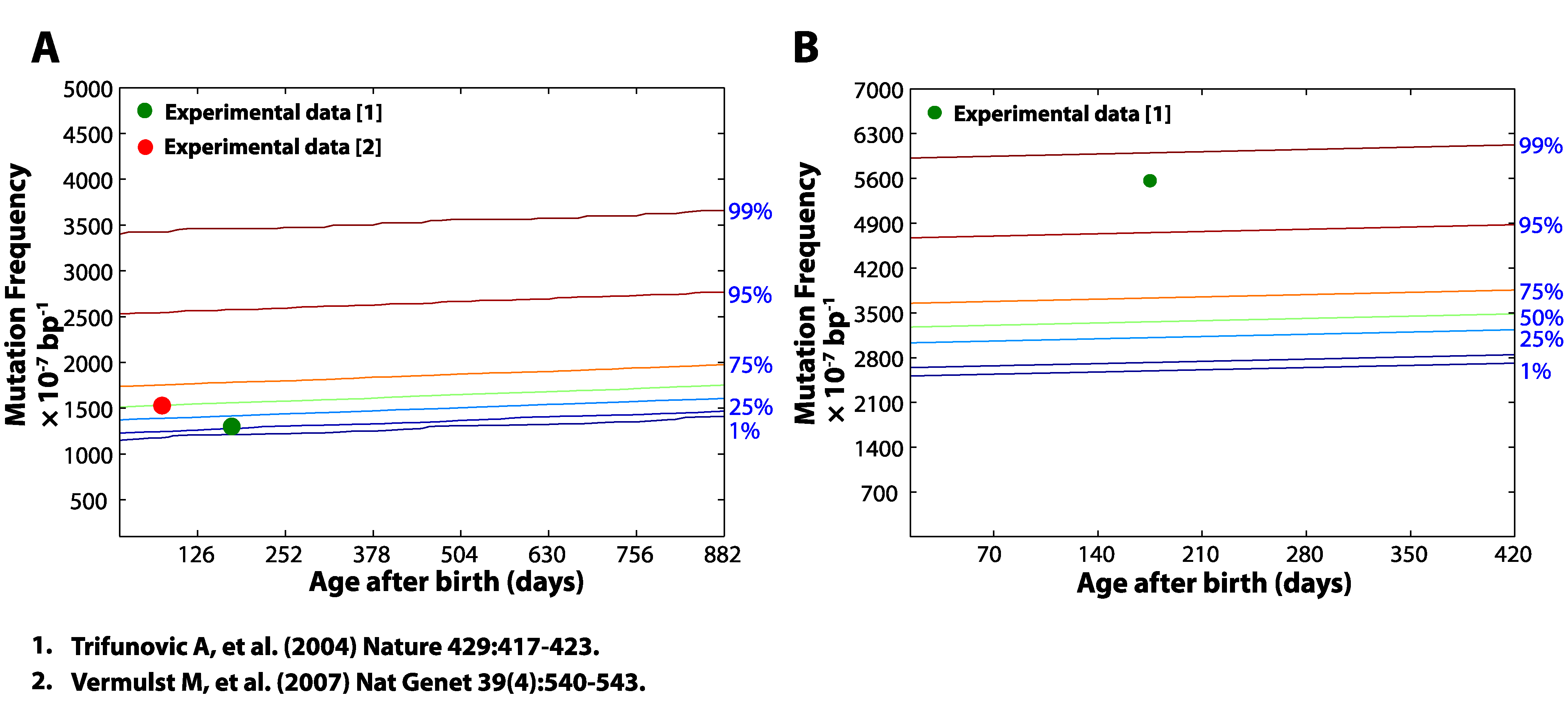

Supplement: Figure S4 — Stochastic determinants of age-dependent point mutation dynamics in mutator mice. Comparison of the observed mtDNA point mutation frequency in a population of 1,100 in silico POLG mouse heart tissues (Heterozygous and Homozygous). The mutation frequencies were recorded every fortnight up to 36 months (heterozygous) and 14 months (homozygous). (A) The percentile curves of the mutation frequency in the RMC assay of in silico POLG+/mut mouse (heterozygous) population. The apparent variability arises from the genetic variations intrinsic to the aging process and the hypergeometric sampling variability in the RMC protocol (details in the Methods section). (B) The percentile curves of the mutation frequency in the RMC assay of in silico POLGmut/mut mouse (homozygous) population. Unlike the wild-type, the uncertainty arising due to the combined effect of the two sources of variability does not increase with time. The variance remains roughly constant with age and this is primarily due to the high point mutation load prevailing in the cells at birth, which only increases relatively marginally with age. (0.50 MB TIF) [file pcbi.1000572.s004.tif]

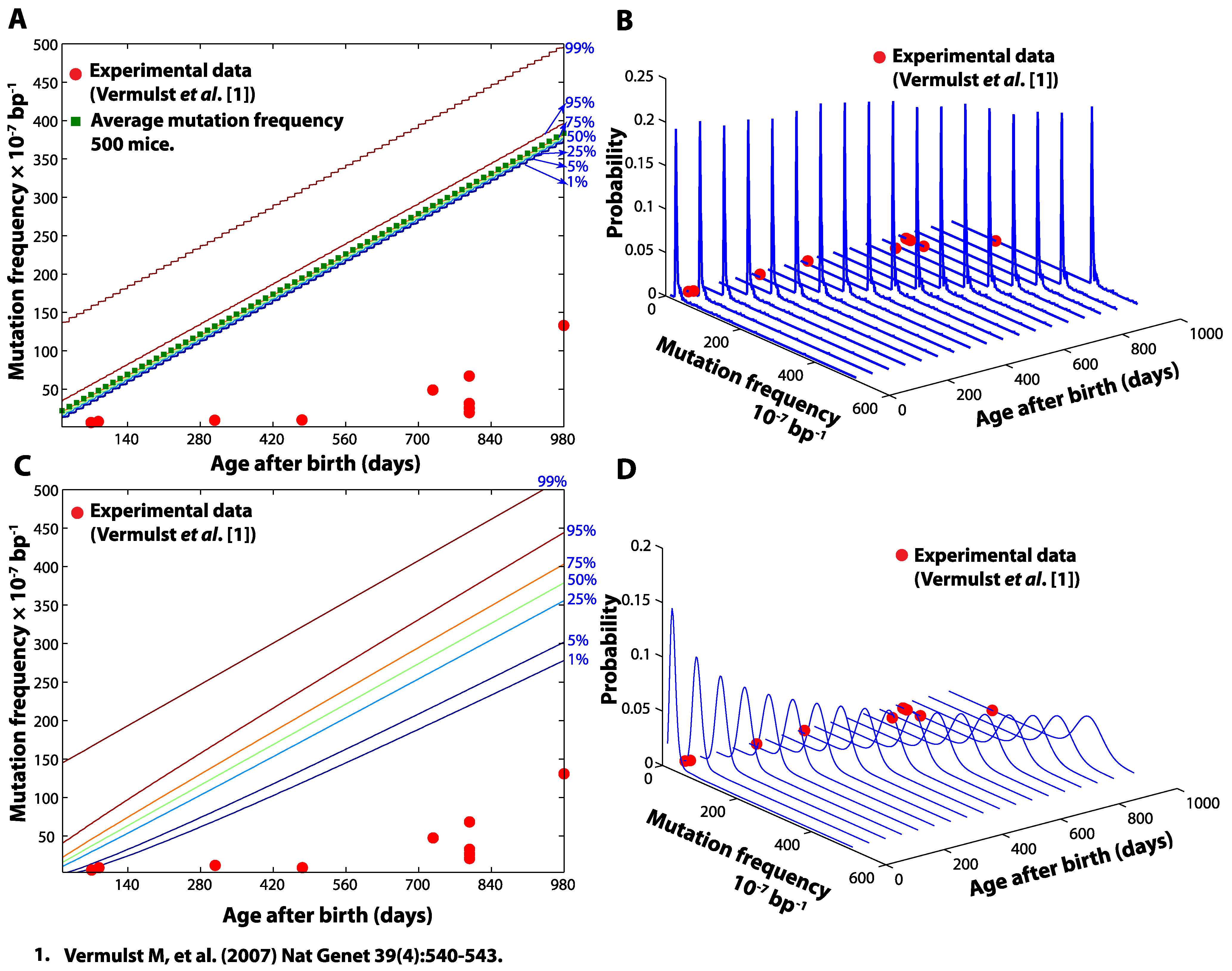

Supplement: Figure S5 — Mitochondrial DNA point mutation burden under an elevated oxidative burden assumption. The oxidative burden used in these simulations was elevated to 10 times higher than that used in the main article (Figure 3 in Main Text). The mutation frequencies were again recorded every fortnight up to 36 months. (A, B) The percentile curve and distribution of the mutation frequency due to the intrinsic stochasticity of aging process in the mouse population (n = 500 mice). (C, D) The percentile curve and distribution of the mutation frequency estimated by the RMC assay of the in silico wild-type mouse population considered. (1.12 MB TIF) [file pcbi.1000572.s005.tif]

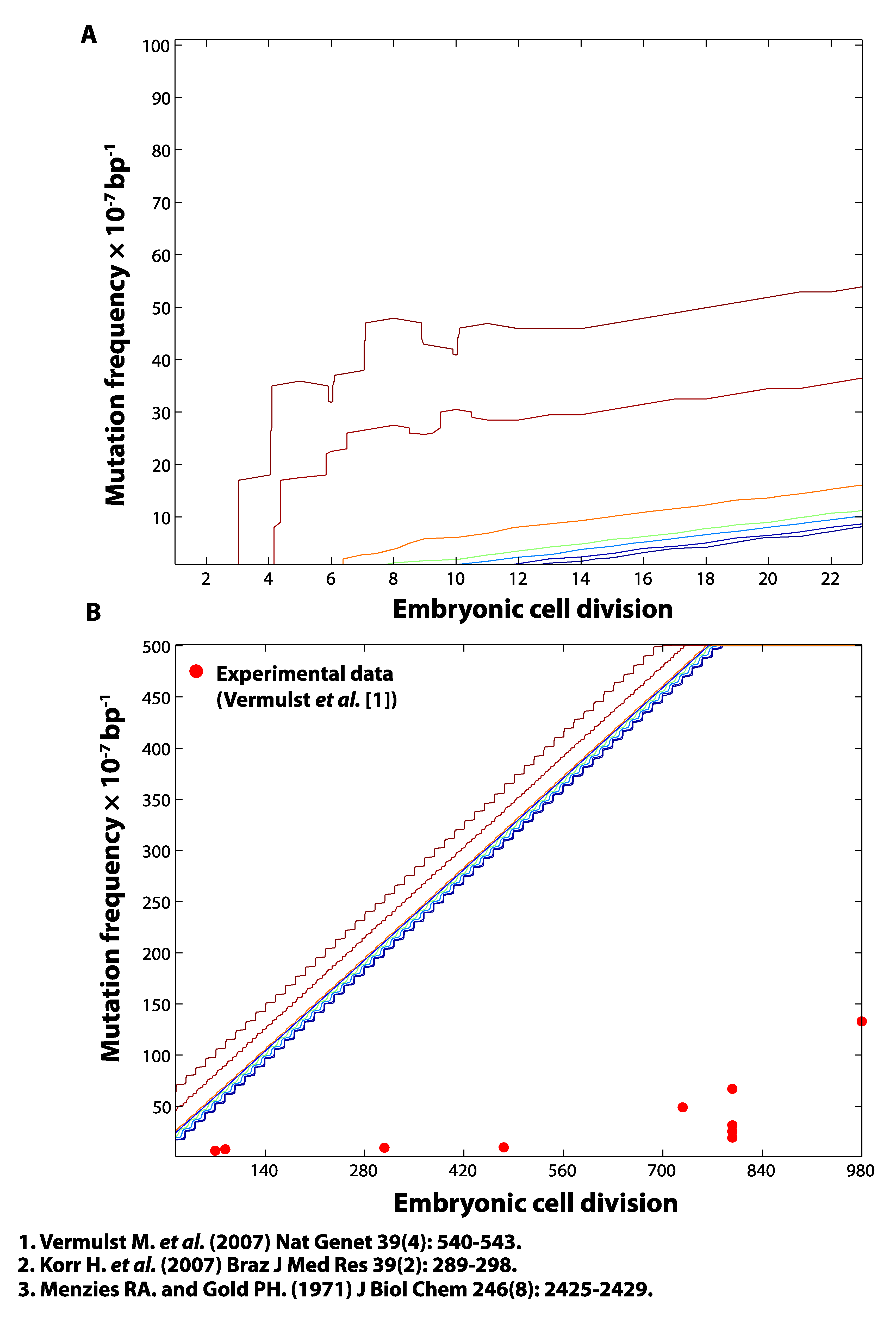

Supplement: Figure S6 — Mitochondrial DNA point mutation burden simulated using mtDNA half-life of 17 days. Inherent mtDNA point mutation frequency in the heart tissues of 100 mice using an mtDNA half life of 17 days (based on the references [2] and [3] indicated in the figure). The mutation frequencies in the post development were monitored every fortnight up to 36 months. (A) Expansion of the mtDNA point mutations during the heart tissue development and, (B) Point mutation dynamics in the mouse population post-birth. (1.23 MB TIF) [file pcbi.1000572.s006.tif]
