# Supplementary material for: Stochastic Drift in Mitochondrial DNA Point Mutations: A Novel Perspective Ex Silico
Source: PLoS Comput Biol. 2009 Nov 20;5(11):e1000572. doi: 10.1371/journal.pcbi.1000572 (PMC2771766; doi:10.1371/journal.pcbi.1000572)
Supplement: Table S3 — Model parameters used in the simulations of the in silico POLG homozygous mice's (POLGmut/mut) mice. (0.07 MB DOC) [file pcbi.1000572.s009.doc]

Table S3: Model parameters used in the simulations of the *in silico* POLG heterozygous mice’s (POLG^mut/mut^) mice

| **Parameters** | **Unit** | **Values** | **Comments** | **References** |
| --- | --- | --- | --- | --- |
| **Development** |  |  |  |  |
| $k_{R}^{dev}$ | *molecules d^-1^* | 465 **(Heart)**  530 **(Liver)** | Maximum replication rate of mtDNA |  |
| *W_0_* | *molecules* | 586 | Initial value of wild type mtDNA | [1,2] |
| *M_0_* | *molecules* | 0 | Initial value of mutant mtDNA |  |
| *k_d_* | *d^-1^* | 2.3377×10^-3^ | Degradation of mtDNA | [3] |
| $k_{m}^{dev}$ | *rep^-1^ bp^-1^* | 1.0×10^-7^ × 200  = 2.0×10^-5^ | POLG fidelity error rate. | [4-6] |
| *N_cyc_* | - | 22 | Number of developmental cycles | [2,7-9] |
| **Postnatal** |  |  |  |  |
| $k_{R}^{PM}$ | *molecules d^-1^* | 0.8182 **(Heart)**  0.9351 **(Liver)** | Maximum replication rate of mtDNA |  |
| *(W+M)_ss_* | *molecules* | 3500 **(Heart)**  4000 **(Liver)** | Homeostatic set-point of the mtDNA population | [10,11] |
| $k_{m}^{PM}$ | *rep^-1^ bp^-1^* | 2.17×10^-5^ | Mutation rate of POLG fidelity error rate with oxidative error | [4-6,12-14] |
| *N_cell_* | - | 2.2443×10^7^ **(Heart)**  4.1871×10^8^ **(Liver)** | Number of cells | [11,15] |

1. Elliott K, O'Connor M (1976) Embryogenesis in mammals (Ciba Foundation symposium ; 40): Elsevier.

2. Piko L, Taylor D (1987) Amounts of Mitochondrial DNA and Abundance of Some Mitochondrial Gene Transcripts in Early Mouse Embryos. Dev Bio 123: 364--374.

3. Collins ML, Eng S, Hoh R, Hellerstein MK (2003) Measurement of mitochondrial DNA synthesis in vivo using a stable isotope-mass spectrometric technique. J Appl Physiol 94: 2203--2211.

4. Cervantes RB, Stringer JR, Shao C, Tischfield JA, Stambrook PJ (2002) Embryonic stem cells and somatic cells differ in mutation frequency and type. Proc Natl Acad Sci USA 99(6): 3586--3590.

5. Kunkel TA (1992) DNA Replication Fidelity. J Biol Chem 267(26): 18251--18254.

6. Zhang D, Mott JL, Chang SW, Denniger G, Feng Z, et al. (2000) Construction of Transgenic Mice with Tissue-Specific Acceleration of Mitochondrial DNA Mutagenesis. Genomics 69: 151-161.

7. Larsson NG, Wang J, Wilhelmsson H, Oldfors A, Rustin P, et al. (1998) Mitochondrial transcription factor A is necessary for mtDNA maintenance and embryogenesis in mice. Nat Genet 18: 231--236.

8. Karatza C, Stein WD, Shall S (1984) Kinetics of in vitro ageing of mouse embryo fibroblasts. J Cell Sci 65: 163-175.

9. Sissman NJ (1970) Developmental Landmarks in Cardiac Morphogenesis: Comparative Chronology. Am J Cardiol 25: 141--148.

10. Weiss JN (1997) The Hill equation revisited: uses and misuses. FASEB 11: 835--841.

11. Wiesner RJ, Ruegg JC, Morano I (1992) Counting target molecules by exponential polymerase chain reaction: copy number of mitochondrial DNA in rat tissue. Biochem Bioph Res Co 183(2): 553--559.

12. Cadet ARJ, Cadet J, Moller L, Poulsen HE, Vina J (2004) Are we sure we know how to measure 8-oxo-7,8-dihydroguanine in DNA from human cells? Arch Biochem Biophys 423(1): 57--65.

13. ESCODD (2002) Inter-laboratory validation of procedures for measuring 8-oxo-7,8-dihydroguanine/8-oxo-7,8-dihydro-2'-deoxyguanosine in DNA. Free Radic Res 36: 239--245.

14. ESCODD (2002) Comparative analysis of baseline 8-oxo-7,8-dihydroguanine in mammalian cell DNA, by different methods in different laboratories: an approach to consensus. Carcinogenesis 23: 2129--2133.

15. Limson M, Jackson CM (1931) Changes in the weights of various organs and systems of young rats maintained on a low-protein diet. J Nutr 5(2): 163--174.
