# Supplementary material for: Stochastic Drift in Mitochondrial DNA Point Mutations: A Novel Perspective Ex Silico
Source: PLoS Comput Biol. 2009 Nov 20;5(11):e1000572. doi: 10.1371/journal.pcbi.1000572 (PMC2771766; doi:10.1371/journal.pcbi.1000572)
Supplement: Text S1 — Supporting Information (0.07 MB DOC) [file pcbi.1000572.s010.doc]

**ST.1 Computation of mtDNA mutation frequency**

The point mutation burden (mutation frequency) per base pair is determined using,

$\Delta_{f}^{sim}=\frac{M_{tot}}{\left(W_{tot}+M_{tot}\right)\cdot4bp}$ [1]

where *W_tot_* and *M_tot_* are the total number of wild-type and mutant mtDNA molecules in the tissue, respectively. The length of *Taq*I recognition site used in the RMC assay is 4 bp [1]. Note that the probability of a molecule with two or more mutations in the same *Taq*I site is negligible.

**ST.2 Replication models of mtDNA**

The mtDNA copy number is maintained roughly constant throughout the cell growth and divisions [2]. The degraded mtDNA are continuously replaced with new ones and mtDNA replication should occur as much as their degradation. There exist evidence indicating the existence of a retrograde signaling between mitochondria and nucleus to regulate the mtDNA content based on cellular bioenergetics [3]. This suggests that mitochondrial biogenesis may be initiated as soon as the mtDNA copy numbers in a cell falls below a certain homeostatic set-point value. For the present work we have explored two models of mtDNA replication a.) constant biogenesis and b.) biogenesis with retrograde signaling. In the first model, a constant mtDNA replication rate was deduced based on the homeostatic mtDNA copy number in a cell and the degradation rate of mtDNA above. In this case, the replication propensity is given by:

$a_{R}=k_{d} \cdot\left(W+M\right)_{ss}$ [2]

where $\left(W+M\right)_{ss}$ represents the homeostatic level of mtDNA population in the cell. In the second model, a Hill-type cooperative equation was used to simulate the retrograde signaling. The Hill-type equation has been widely used in the modeling of biological system involving switch-like behavior arising from cooperativity of enzymes [4]. The functional effect of mutations is simulated as a retrograde signaling by means of a Hill-type kinetics, insofar as the Hill-feedback responds to a drop in the number of wild-type (and hence functional) mtDNA. In other words, a mutant mtDNA is considered to be entirely dysfunctional. Notice that the maximum rate of replication by the Hill-type kinetics is twice that of the constant biogenesis parameter. Using the Hill-feedback, our simulations show that wild-type mtDNA population in a cell rarely drops large enough to trigger an increased replication by this feedback and the simulations of the two model assumptions are in agreement as shown in the Supplementary Figure S3 (bar plot b). The parameters of the Hill-type equation, *K_H_* and *n* can be used as a set point level of mtDNA numbers and the sharpness of the switch response, respectively. The replication propensity is then a composite of: a.) maximum replication rate, balancing the degradation rate of mtDNA, and b.) negative Hill term capturing the retrograde response of the nucleus. Hence, the replication propensity for replication of mtDNA is given by:

$a_{R}=\nu_{\max}\cdot\left(1-\left\{\frac{W^{n}}{K_{H}^{n}+W^{n}}\right\}\right)$ [3]

where $\nu_{\max}$ is the maximum replication rate computed by,

$\nu_{\max}=\frac{k_{d}\cdot K_{H}}{0.5}$ [4]

Due to the low frequency of the point mutation, the difference between the two replication models is negligible (Figure S3). We have presented the constant biogenesis replication model, in the main text, as this minimizes the number of model parameters and assumptions.

**ST.3 Selection of turnover rate.**

Literature values regarding mitochondrial turnover differ widely, citing half life values ranging from 6 days to ~300 days [5-9]. The literature is relatively sparse and spans many decades. Consequently the methods utilized and the tissues examined differ significantly between studies, making direct comparison somewhat difficult. Irrespective of whether protein or DNA turnover is monitored, turnover studies usually rely on efficient separation of mitochondria from tissue. However, we have found in our own work [10] that most of the standard techniques, including differential centrifugation, do not reliably purify mitochondria to a sufficiently high degree of purity (as judged by PCR detection of significant nDNA contamination in mitochondrial pellets). Protein labeling studies have additional complexity as the label (e.g.: ^14^C NaHCO_3_) is first metabolized and incorporated into protein, consequently the interpretation of results relies on a detailed understanding of the fate of the label in different tissues. An additional concern is that protein turnover is not necessarily dependent on turnover of the whole mitochondrion [9].

For the context of this study mtDNA turnover maybe the most relevant and we have found only one publication that examines this and also proves purity of mtDNA by PCR [8]. We have consequently utilized this value for our simulations as the current “best guess” for mtDNA turnover. However, we do appreciate that turnover rates are commonly reported (e.g. by Korr *et al.* [5] and Menzies et al. [6]) at considerably higher values than those assumed in our model. In addition to the above argument regarding experimental technique, we feel that, using a relatively simple analytical approximation for the expected mutation burden, the point can be made that these higher turnover values are unlikely, at least in a first approximation.

If we consider a half life of 17 days as suggested by [5,6], the number of mtDNA molecules turned over daily in a typical cardiomyocyte with 3500 mtDNA molecules is:

$$\frac{\ln2}{\text{17days}}\times3500 \approx143 molecules/day$$

During the life-span of a mouse (~ 3 years), the total number of mtDNA molecule copying events then comes to 156,585. Considering the fact that mouse mtDNA has 16,565 bps, this means that in total almost 2.6 x 10^9^ bps are copied. Assuming a basal error rate of $1\times10^{-6}$per bp copied, this means that at the end of life almost 2600 *de novo* point mutations would have occurred in each cell, corresponding to a mutation rate of almost 4.5 x10^-5^ per bp – a level that is inconsistent with the values observed in RMC data. We further simulated 100 mouse hearts with a higher turnover rate of 0.041 day^-1^ (corresponding to the half life of 17 days), while keeping all the other parameters the same. The result of this analysis is shown in Figure S6.

In general, we find that unless there could be a preferential degradation of mutant against the wild-type mtDNA (which could be envisioned for functionally relevant mtDNA mutations), the higher turnover rate gives mutation frequencies that are much higher than actually identified by the RMC assay [5]. A shorter half-life will definitely give an even higher mutation frequency prediction. While we plan to explore preferential degradation in the future, this is likely to be dependent on some degree on compartmentalization (fusion, fission dynamics), retrograde signaling and functional threshold effects, and will therefore add a significant level of complexity (and parameters) to the model. Even with the simplifications made, the current model has allowed us to better understand some of the more surprising results and opaque factors in the mutator mouse data and believe that one of its strengths is its parsimony and the low number of parameters.

**ST.4 References**

1. Vermulst M, Bielas JH, Kujoth GC, Ladiges WC, Rabinovitch PS, et al. (2007) Mitochondrial point mutations do not limit the natural lifespan of mice. Nat Genet 39(4): 540--543.

2. Davis AF, Clayton DA (1996) In Situ Localization of Mitochondrial DNA Replication in Intact Mammalian Cells. J Cell Biol 135: 883--893.

3. Liu Z, Butow RA (2006) Mitochondrial retrograde signaling. Annu Rev Genet 40: 159--185.

4. Weiss JN (1997) The Hill equation revisited: uses and misuses. FASEB 11: 835--841.

5. Korr H, Kurz C, Seidler TO, Sommer D, Schmitz C (1998) Mitochondrial DNA synthesis studied autoradiographically in various cell types in vivo. Braz J Med Biol Res 31: 289-298.

6. Menzies RA, Gold PH (1971) The turnover of mitochondria in a variety of tissues of young adult and aged rats. J Biol Chem 246: 2425-2429.

7. Gross NJ, Getz GS, Rabionowitz M (1969) Apparent turnover of mitochondrial Deoxyribonucleic acid and mitochondrial phospholipids in the tissues of the rat. J Biol Chem 244(6): 1552--1562.

8. Collins ML, Eng S, Hoh R, Hellerstein MK (2003) Measurement of mitochondrial DNA synthesis in vivo using a stable isotope-mass spectrometric technique. J Appl Physiol 94: 2203--2211.

9. Miwa S, Lawless C, von Zglinicki T (2008) Mitochondrial turnover in liver is fast in vivo and is accelerated by dietary restriction: application of a simple dynamic model. Aging Cell 7: 920-923.

10. Lim KS, Jeyaseelan K, Whiteman M, Jenner A, Halliwell B (2005) Oxidative damage in mitochondrial DNA is not extensive. Ann N Y Acad Sci 1042: 210--220.
