# Supplementary material for: Stochastic Drift in Mitochondrial DNA Point Mutations: A Novel Perspective Ex Silico
Source: PLoS Comput Biol. 2009 Nov 20;5(11):e1000572. doi: 10.1371/journal.pcbi.1000572 (PMC2771766; doi:10.1371/journal.pcbi.1000572)
Supplement: Text S2 — Stochastic mtDNA Point Mutation Simulation Algorithm (0.03 MB PDF) [file pcbi.1000572.s011.pdf]

```
C########################################################################################################################################################
C########################################################################################################################################################
C   $$$$$$$$$$$$$$$$$$$$$$$$$$$$$$$$$$$$$$$$$$$$$$$    mtDNA POINT MUTATION ALGORITHM FOR MICE   $$$$$$$$$$$$$$$$$$$$$$$$$$$$$$$$$$$$$$$$$$$$$$$$$$$$$$$$
C################################
C       PURPOSE
C###############################
C       THIS ALGORITHM IS FOR SIMULATING THE EVOLUTION mtDNA POINT MUTATION DURING BOTH THE DEVELOPMENTAL AND POST BIRTH STAGES OF MICE. DIFFERENT MICE
MODELS CAN BE SIMULATED BY CHANGING THE INPUT PARAMETERS...

C################################
C       GENERAL DETAILS
C###############################

C       $$$$$$$$$$$$$$$$$$$$$$$$$$$$$$$$$$$$$$$$$$$$$$$         STATISTICS RUN ALGORITHM        $$$$$$$$$$$$$$$$$$$$$$$$$$$$$$$$$$$$$$$$$$$$$$$$$$$$$$$$$

C       Modified Stochastic algorithm (SSA IMPLEMENTATION) for understanding the evolution of the Wild/Mutant mt-DNA molecules.
C       The replication and degradation processes are simulated to occur in a batch of 10, as during the replication or degradation stages the entire
C       mitochondria, which comprises of about 2-10 mt-DNA molecules is subjected to the either of the processes.

C       The developmental stage comprsises of simulation of 22 cell dvisions, where the mitochondrial DNA is randomly picked from the population and
C       subjected to both replication and degradation until the population size of mtDNA approximately doubles.

C       At the end of 22 cycles of cell division in the embryonic development, the mtDNA of the differentiated cells undergo relaxed replication till
the
C       end of mice's life span (3 years)

C       The CME reaction considered for the studies are,
C       Mutation of the Wild type molecules,
C                                               W -----> W + M          (rate = c1 frequency)
C               The mutation of the wild type is considered to be occuring during the stage of replication of the wild type molecules, where the
C               replicating molecule has a certain probability to undergo mutation, characterized by the mutation rate.
C                                               a1 = c1*c4*(1-W^n/(K^n + W^n))
C       Degradation of the Wild type mt-DNA,
C                                               W -----> 0              (rate = c2 day^{-1})
C       Degradation of the mutant type mt-DNA,
C                                               M -----> 0              (rate = c3 day^{-1})
C       Replication of the mt-DNA molecules,
C                                               W|M ------> 2w|2M       (rate = c4 molecules.day^{-1})
C               The Propensity of the replication of the mt-DNA molecules is given as,
C                                               a4 =  c2|c3*(W+M)_SS
C-------------------------------------------------------------------------------------------------------------------------------------------------------
C       Created by : Suresh Poovathingal, NUS, Singapore
C       Modified by : Suresh Poovathingal
C       Last Modified on : 17/07/2009
C-------------------------------------------------------------------------------------------------------------------------------------------------------
C       contact : suresh.poovathingal@nus.edu.sg

C########################################################################################################################################################

        PROGRAM AgingTrialAlgSSAStats

C       Delcaration of various Variables used in this code
        DOUBLE PRECISION DW,DM,C1,C2,C4,HILLN,HILLK,A(4),ATOTAL,TAU,COMPARE,AADD
        DOUBLE PRECISION URN1,URN2
        DOUBLE PRECISION CURN
        DOUBLE PRECISION SIMTIME,STARTW,STARTM
```

```
      DOUBLE PRECISION RURN,SMOLRATIO
      DOUBLE PRECISION RMURN,TIMECOUNT
      DOUBLE PRECISION DURN
      DOUBLE PRECISION TOTMITO,SUMSTATES,SUMHALF,SUMMUT,XMRET,HGURN,SUMWILD_DEV,SUMMUTANT_DEV,SUMWILD_POSTDEV,SUMMUTANT_POSTDEV
      DOUBLE PRECISION CM,RMSURN
      DOUBLE PRECISION HILLK_PD,CPM,AMUTFREQ(6)
      DOUBLE PRECISION SWILDTEMP(200),SMUTTEMP(200),TIME_INTERVAL(6),DW_TEMP,DM_TEMP,SAMPLE_INTER_WILD(200),SAMPLE_INTER_MUTANT(200)
      DOUBLE PRECISION TIME_INC,TEMP_TIME

      INTEGER ICURN
      INTEGER IDUMC,IDUMC2,IVC(32),IYC
      INTEGER IDUM1,TIME,IDUM12,IV1(32),IY1,IDUM2,IDUM22,IV2(32),IY2,ISTCNT,IACOUNT,NXTRCNT,ITOTSTAT
      INTEGER IRDUM,IRDUM2,IRV(32),IRY,IRSEED,IRCOUNT,ISEEDVAL,ISEEDVALR
      INTEGER IMDUM,IMDUM2,IMV(32),IMY,IMSEED,IWRITE
      INTEGER IDUMD,IDUMD2,IVD(32),IYD
      INTEGER IDCOUNT,IDSEED
      INTEGER ICELLCYCLE,ICYCLETIME,IOCOUNT,ICYCLE,NCYCLE,INCOUNT
      INTEGER NNLAYER(5000000,2),NOLAYER(5000000,2)
      INTEGER ISUMWILD,ISUMMUTANT
      INTEGER IHGDUM,IHGDUM2,IHGV(32),IHGY,IHBUDUM,IHBUCOUNT,IHGDUM1
      INTEGER IMSDUM,IMSDUM2,IMSV(32),IMSY,IMSSEED
      INTEGER IVAL,IEA
      INTEGER ISAMPLER_COUNT

      CHARACTER ARG*80

C     Delcaration of various constants used in this code
      DATA NNLAYER /10000000*0/,NOLAYER /10000000*0/
      DATA IDUM12 /0/,IDUM22 /0/,IV1 /32*0/,IV2 /32*0/,IY1 /0/,IY2 /0/
      DATA DW /0.0/, DM /0.0/
      DATA IDUMC2 /0/,IVC /32*0/,IYC /0/
      DATA IRDUM2 /0/,IRV /32*0/,IRY /0/
      DATA IMDUM2 /0/,IMV /32*0/,IMY /0/
      DATA IDUMD2 /0/,IVD /32*0/,IYD /0/
      DATA A /4*0.0/
      DATA ICELLCYCLE /0/, ICYCLE /1/, NCYCLE /22/
      DATA INEWCOUNT /1/
      DATA IHGDUM2 /0/,IHGV /32*0/,IHGY /0/
      DATA IMSDUM2 /0/,IMSV /32*0/,IMSY /0/
      DATA SAMPLE_INTER_WILD /200*0.D0/,SAMPLE_INTER_MUTANT /200*0.D0/

C##########################################################################################################################################
C     Data input from the external file (Rate constants for the simulation of mouse models)

      OPEN(UNIT=59,FILE='CellCicleInput_POLGHetero_Complete',FORM='FORMATTED')
      READ(UNIT=59,FMT=201)HILLN,HILLK         ! Parameters of the Hill equation for developmental stage (n, K_dev)
201   FORMAT(F10.2,F10.2)
      READ(UNIT=59,FMT=209)HILLK_PD            ! Parameters of the Hill equation for post-developmental stage (K_post dev)
209   FORMAT(F10.2)
      READ(UNIT=59,FMT=202)SIMTIME             ! Total simulation time (age of mice = 3 years)
202   FORMAT(F10.2)
      READ(UNIT=59,FMT=203)C2,C3               ! mtDNA degradation rate constant (k_d)
203   FORMAT(F10.8,1X,F10.8)
      READ(UNIT=59,FMT=206)CC                  ! mtDNA replication rate for the developmental stage (k_R(dev))
206   FORMAT(F11.2)
      READ(UNIT=59,FMT=208)CPM                 ! mtDNA replication rate for the post developmental stage (k_R(PM))
208   FORMAT(F11.5)
      READ(UNIT=59,FMT=207)C1,CM               ! mtDNA mutation rate per replication of mtDNA in the developmental stage (k_m(dev))
```

```
207     FORMAT(F12.10,1X,F12.10)
        READ(UNIT=59,FMT=210)C1_PD,CM_PD        ! mtDNA mutation rate per replication of mtDNA in the post-mitotoc stage (k_m(PM))
210     FORMAT(F12.10,1X,F12.10)
        READ(UNIT=59,FMT=204)STARTW,STARTM      ! mtDNA initial count for wild-type and mutant at the start of development (W_0, M_0)
204     FORMAT(F9.2,F9.2)
        READ(UNIT=59,FMT=205)ITOTSTAT           ! Number of stages of development
205     FORMAT(I9)
        CLOSE(UNIT=59)

C       Certain inputs which have two readings per input are specifically for the modeling POLG heterozygous mice (POLG +/mut), representing kinetic
rate
C       constaants of the two alleles

C###########################################################################################################################################################

C       $$$$$$$$$$$$$$$$$$$$$$$$$$$$$$$$$$$$$$$$$$$$$$$$$$$$$$$         MAIN PROGRAM    $$$$$$$$$$$$$$$$$$$$$$$$$$$$$$$$$$$$$$$$$$$$$$$$$$$$$$$$$$$

C###########################################################################################################################################################

C       Command line inputs
        NARGS=IARGC()
        DO IEA=1,NARGS
                CALL GETARG(IEA,ARG)
                READ(ARG,'(I14)')IVAL
        ENDDO

C       $$$$$$$$$$$$$$$$$$$$$$$$$$$$$$$$$$$$$$$$$$$$$$$$$$$$$$$   DEVELOPMENTAL STAGES   $$$$$$$$$$$$$$$$$$$$$$$$$$$$$$$$$$$$$$$$$$$$$$$$$$$$$$$$$$$$$$
        ISTCNT=1
C       Calling the system time for seeding
        ISEEDVALR=TIME()
        IDUMC=-ISEEDVALR-IVAL

C       Reading the initial values of mtDNA states of wild type and mutant
        ICYCLETIME=2**ICELLCYCLE
        IDW=ANINT(STARTW)
        DO 11 IOCOUNT=1,ICYCLETIME
                NOLAYER(IOCOUNT,1)=IDW
                WRITE(*,200)DBLE(NOLAYER(IOCOUNT,1)),DBLE(NOLAYER(IOCOUNT,2))
11      CONTINUE

C       DEVELOPMENTAL CYCLES
        DO WHILE(ICYCLE.LE.NCYCLE)
                INCOUNT=1
                IHBUCOUNT=1     ! Counter for seeding Hypergeometric Distribution
                !  Number of cell division in the present stage
                DO IOCOUNT=1,ICYCLETIME
                        DW=DBLE(NOLAYER(IOCOUNT,1))     ! Input of the wild type anr mutant mtDNA
                        DM=DBLE(NOLAYER(IOCOUNT,2))

C                       **************   Seeding scheme for independent uniform random number streams **********************
                        ISEEDVAL=TIME()
                        ISEEDVAL=ISEEDVAL+IVAL
                        CALL RANDOM(IDUMC,IDUMC2,IVC,IYC,CURN)
                        CURN=CURN*100000000
                        ICURN=IDINT(CURN)
                        IF(ICURN.EQ.0)THEN
                                ISEEDVAL=ISEEDVAL+ICYCLETIME
                                IDUM1=-ISEEDVAL
```

```
                              ELSE
                                      IDUM1=-ISEEDVAL-ICURN-ICYCLETIME
                              ENDIF
                              IDUM2=IDUM1-56
                              IRSEED=IDUM1
                              IMSEED=IDUM1
                              IDSEED=IDUM1
                              IHGSEED=IDUM1
                              IMSSEED=IDUM1
                              IRDUM=IRSEED-115
                              IMDUM=IMSEED-210
                              IDUMD=IDSEED-460
                              IHGDUM=IHGSEED-900
                              IHGDUM1=IHGSEED-900
                              IMSDUM=IMSEED-690
C                             **************   End of scheme  ***********************

                              !  SSA initializations
                              TOTMITO=HILLK
                              SUMSTATES=0.0
                              TIMECOUNT=0.0
                              !  SSA implementation per cell cycle
                              DO WHILE(SUMSTATES.LT.TOTMITO)
                                      IHBUDUM=IHGDUM1         !  seeding Hypergeometric Distribution

C                                     Propensity function definitions...
                                      A(1)=C2*(DW+DM)
C                                     A(2)=CC*(1-(((DW+DM)**HILLN)/((HILLK**HILLN)+((DW+DM)**HILLN))))
                                      A(2)=CC
                                      ATOTAL=0.0
                                      DO 10 IACOUNT=1,2
                                              ATOTAL=ATOTAL+A(IACOUNT)
10                                    CONTINUE

C                                     Uniform random number generations for the SSA implementation
                                      CALL RANDOM(IDUM1,IDUM12,IV1,IY1,URN1)
                                      CALL RANDOM(IDUM2,IDUM22,IV2,IY2,URN2)

C                                     SSA tau leap detemination
                                      TAU=(1.0/ATOTAL)*LOG(1.0/URN1)
                                      TIMECOUNT=TIMECOUNT+TAU

C                                     SSA next reaction event locator
                                      COMPARE=URN2*ATOTAL
                                      NXTRCNT=0
                                      AADD=0.0
                                      DO WHILE(AADD.LT.COMPARE)
                                              NXTRCNT=NXTRCNT+1
                                              AADD=AADD+A(NXTRCNT)
                                      ENDDO

C                                     SSA reaction stoichimetry update
                                      IF(NXTRCNT.EQ.1)THEN    ! Degradation reaction
                                              DO 19 IDCOUNT=1,10
                                                      SMOLRATIO=DW/(DW+DM) ! Selection based on the ratio of states
                                                      CALL RANDOM(IDUMD,IDUMD2,IVD,IYD,DURN)
                                                      IF((DURN.LE.SMOLRATIO).AND.(DW.GT.0.0))THEN
                                                              DW=DW-1.
```

```
                                        ELSEIF(DM.GT.0.0)THEN
                                                DM=DM-1.
                                        ENDIF
19                              CONTINUE
                        ELSEIF(NXTRCNT.EQ.2)THEN        ! Replication reaction
                                DO 15 IRCOUNT=1,10
                                        SMOLRATIO=DW/(DW+DM)    ! Selection based on the ratio of states
                                        CALL RANDOM(IRDUM,IRDUM2,IRV,IRY,RURN)
                                        IF(RURN.LE.SMOLRATIO)THEN
                                                CALL RANDOM(IMDUM,IMDUM2,IMV,IMY,RMURN)
                                                CALL RANDOM(IMSDUM,IMSDUM2,IMSV,IMSY,RMSURN) ! Frequecy check for mtDNA mutation
                                                IF(RMSURN.LT.0.5D0)THEN
                                                        SMC=C1
                                                ELSE
                                                        SMC=CM
                                                ENDIF
                                                IF(RMURN.LE.SMC)THEN
                                                        DM=DM+1.

                                                ELSE
                                                        DW=DW+1.

                                                ENDIF
                                        ELSE
                                                DM=DM+1.
                                        ENDIF
15                              CONTINUE
                        ENDIF
                        SUMSTATES=DW+DM
                ENDDO

C               Seggregation of the mtDNA states between the dividing cells
                ITEMP=(2*INCOUNT)-1
                IF(DM.EQ.0.0D0)THEN             ! Case of no mutants
                        SUMHALF=ANINT(SUMSTATES/2.D0)
                        NNLAYER(ITEMP,2)=0
                        NNLAYER(ITEMP,1)=ANINT(SUMHALF)
                        NNLAYER((ITEMP+1),2)=INT(DM-NNLAYER(ITEMP,2))
                        NNLAYER((ITEMP+1),1)=INT(DW-NNLAYER(ITEMP,1))
                ELSEIF(DW.EQ.0.0D0)THEN         ! Case of no wild-type mtDNA
                        SUMHALF=ANINT(SUMSTATES/2.D0)
                        NNLAYER(ITEMP,2)=ANINT(SUMHALF)
                        NNLAYER(ITEMP,1)=0
                        NNLAYER((ITEMP+1),2)=INT(DM-NNLAYER(ITEMP,2))
                        NNLAYER((ITEMP+1),1)=INT(DW-NNLAYER(ITEMP,1))
                ELSE                            ! Case of hypergeometric seggrergation of mutant mtDNA between the dividing daughter cells
                        SUMHALF=ANINT(SUMSTATES/2.D0)
                        SUMMUT=DM
                        CALL RANDOM(IHGDUM,IHGDUM2,IHGV,IHGY,HGURN)
                        IHBUDUM=IHBUDUM-IHBUCOUNT
                        CALL HYPERGDIST(SUMHALF,SUMMUT,SUMSTATES,HGURN,IHBUDUM,XMRET)
                        IHBUCOUNT=IHBUCOUNT+1
                        NNLAYER(ITEMP,2)=ANINT(XMRET)
                        NNLAYER(ITEMP,1)=ANINT(SUMHALF-NNLAYER(ITEMP,2))
                        NNLAYER((ITEMP+1),2)=INT(DM-NNLAYER(ITEMP,2))
                        NNLAYER((ITEMP+1),1)=INT(DW-NNLAYER(ITEMP,1))
                ENDIF
```

```
                INCOUNT=INCOUNT+1
        ENDDO
        ICELLCYCLE=ICELLCYCLE+1
        ICYCLETIME=2**ICELLCYCLE
        SUMWILD_DEV=0.D0
        SUMMUTANT_DEV=0.D0
        DO 25 ITRANSCNT=1,ICYCLETIME
                NOLAYER(ITRANSCNT,1)=NNLAYER(ITRANSCNT,1)
                NOLAYER(ITRANSCNT,2)=NNLAYER(ITRANSCNT,2)
                SUMWILD_DEV=SUMWILD_DEV+NNLAYER(ITRANSCNT,1)
                SUMMUTANT_DEV=SUMMUTANT_DEV+NNLAYER(ITRANSCNT,2)
25      CONTINUE
        WRITE(*,200)SUMWILD_DEV,SUMMUTANT_DEV
        ICYCLE=ICYCLE+1
C       write(*,*)ICYCLE
    ENDDO
    MAXCELL=2**(ICYCLE-1)

C   $$$$$$$$$$$$$$$$$$$$$$$$$$$$$$$$$$$$$$$$$$$$$$$$$$$$$$$$$$$   POST DEVELOPMENTAL STAGES     $$$$$$$$$$$$$$$$$$$$$$$$$$$$$$$$$$$$$$$$$$$$$$$$$$$$$$$$$$$
c   Random number counter re-initializations
    IDUMC2=0
    IDUM12=0
    IDUM22=0
    IDUMD2=0
    IRDUM2=0
    IMSDUM2=0
    DO 12 ICOUNTER=1,32
        IVC(ICOUNTER)=0
        IV1(ICOUNTER)=0
        IV2(ICOUNTER)=0
        IVD(ICOUNTER)=0
        IRV(ICOUNTER)=0
        IMSV(ICOUNTER)=0
12  ENDDO
    IYC=0
    IY1=0
    IY2=0
    IYD=0
    IMY=0
    IMSY=0

C   Calling the system time for seeding
    ISTCNT=1
    ISEEDVALR=TIME()
    IDUMC=-ISEEDVALR-IVAL

C   Post natal simulation of the mtDNA relaxed replication
    ITOTSTAT=MAXCELL
    DO WHILE(ISTCNT.LE.ITOTSTAT)

        DW=DBLE(NNLAYER(ISTCNT,1)) ! Input of the wild type and mutant mtDNA of cell and the end of developmental stage
        DM=DBLE(NNLAYER(ISTCNT,2))

C       **************   Seeding scheme for independent uniform random number streams ***********************
        ISEEDVAL=TIME()
        ISEEDVAL=ISEEDVAL+IVAL
        CALL RANDOM(IDUMC,IDUMC2,IVC,IYC,CURN)
```

```
                CURN=CURN*100000000
                ICURN=IDINT(CURN)
                IF(ICURN.EQ.0)THEN
                        ISEEDVAL=ISEEDVAL+ISTCNT
                        IDUM1=-ISEEDVAL
                ELSE
                        IDUM1=-ISEEDVAL-ICURN-ISTCNT
                ENDIF
C               IDUM1=-ISEEDVAL-ISTCNT
                IDUM2=IDUM1-56
                IRSEED=IDUM1
                IMSEED=IDUM1
                IDSEED=IDUM1
                IRDUM=IRSEED-115
                IMDUM=IMSEED-210
                IDUMD=IDSEED-460
C               **************   End of scheme ***********************

C               Initializations for intermitant sampling of the mtDNA states
                TIME_INC=14.D0
                TEMP_TIME=14.D0

                !  SSA initializations
                TIMECOUNT=0.0D0
                ISAMPLE_ITERATOR=1
                !  SSA implementation per cell
                DO WHILE(TIMECOUNT.LE.SIMTIME)
                        !  Propensity function definitions...
                        A(1)=C2*(DW+DM);
C                       A(2)=C4*(1-(((DW+DM)**HILLN)/((HILLK**HILLN)+((DW+DM)**HILLN))))
                        A(2)=CPM
                        ATOTAL=0.0D0
                        DO 13 IACOUNT=1,2
                                ATOTAL=ATOTAL+A(IACOUNT)
13                      CONTINUE

C                       Uniform random number generations for the SSA implementation
                        CALL RANDOM(IDUM1,IDUM12,IV1,IY1,URN1)
                        CALL RANDOM(IDUM2,IDUM22,IV2,IY2,URN2)

C                       SSA tau leap detemination
                        TAU=(1.0/ATOTAL)*LOG(1.0/URN1)
                        TIMECOUNT=TIMECOUNT+TAU

C                       SSA next reaction event  locator
                        COMPARE=URN2*ATOTAL
                        NXTRCNT=0
                        AADD=0.0
                        DO WHILE(AADD.LT.COMPARE)
                                NXTRCNT=NXTRCNT+1
                                AADD=AADD+A(NXTRCNT)
                        ENDDO

                        DW_TEMP=0.D0    !  Adjustment of state counters during sampling
                        DM_TEMP=0.D0

C                       SSA reaction stoichimetry update
                        IF(NXTRCNT.EQ.1)THEN            ! Degradation reaction
```

```
                        DO 20 IDCOUNT=1,10
                            SMOLRATIO=DW/(DW+DM)    ! Selection based on the ratio of states
                            CALL RANDOM(IDUMD,IDUMD2,IVD,IYD,DURN)
                            IF((DURN.LE.SMOLRATIO).AND.(DW.GT.0.0))THEN
                                DW=DW-1.0D0
                                DW_TEMP=DW_TEMP-1.D0
                            ELSEIF(DM.GT.0.0)THEN
                                DM=DM-1.0D0
                                DM_TEMP=DM_TEMP-1.D0
                            ENDIF
20                      CONTINUE
                    ELSEIF(NXTRCNT.EQ.2)THEN        ! Replication reaction
                        DO 16 IRCOUNT=1,10
                            SMOLRATIO=DW/(DW+DM)    ! Selection based on the ratio of states
                            CALL RANDOM(IRDUM,IRDUM2,IRV,IRY,RURN)
                            IF(RURN.LE.SMOLRATIO)THEN
                                CALL RANDOM(IMDUM,IMDUM2,IMV,IMY,RMURN)
                                CALL RANDOM(IMSDUM,IMSDUM2,IMSV,IMSY,RMSURN)    ! Frequecy check for mtDNA mutation
                                IF(RMSURN.LT.0.5)THEN
                                    SMC=C1_PD
                                ELSE
                                    SMC=CM_PD
                                ENDIF
                                IF(RMURN.LE.SMC)THEN
                                    DM=DM+1.0D0
                                    DM_TEMP=DM_TEMP+1.D0
                                ELSE
                                    DW=DW+1.0D0
                                    DW_TEMP=DW_TEMP+1.D0
                                ENDIF
                            ELSE
                                DM=DM+1.0D0
                                DM_TEMP=DM_TEMP+1.D0
                            ENDIF
16                      CONTINUE
                    ENDIF

C                   Sampling of mtDNA states intermitantly during the life span of the mice
                    IF(TIMECOUNT.GE.TEMP_TIME)THEN
                        IF(TIMECOUNT.EQ.TEMP_TIME)THEN
                            SWILDTEMP(ISAMPLE_ITERATOR)=DW
                            SMUTTEMP(ISAMPLE_ITERATOR)=DM
                        ELSE
                            SWILDTEMP(ISAMPLE_ITERATOR)=DW-DW_TEMP
                            SMUTTEMP(ISAMPLE_ITERATOR)=DM-DM_TEMP
                        ENDIF
                        TEMP_TIME=TEMP_TIME+TIME_INC
                        ISAMPLE_ITERATOR=ISAMPLE_ITERATOR+1
                    ENDIF
                ENDDO

                DO 79 INIIALIZER=1,(ISAMPLE_ITERATOR-1)
                    SAMPLE_INTER_WILD(INIIALIZER)=SAMPLE_INTER_WILD(INIIALIZER)+SWILDTEMP(INIIALIZER)
                    SAMPLE_INTER_MUTANT(INIIALIZER)=SAMPLE_INTER_MUTANT(INIIALIZER)+SMUTTEMP(INIIALIZER)
79              CONTINUE
                ISTCNT=ISTCNT+1
C               WRITE(*,*)ISTCNT
            ENDDO
```

```
C       Writing out the state values at the corresponding sampling interval
        DO 66 ISAMPCOUNT=1,(ISAMPLE_ITERATOR-1)
                WRITE(*,200)SAMPLE_INTER_WILD(ISAMPCOUNT),SAMPLE_INTER_MUTANT(ISAMPCOUNT)
66      CONTINUE
200     FORMAT(F25.2,1X,F25.2)

        STOP
        END

C###############################################################################################################################################

C       $$$$$$$$$$$$$$$$$$$$$$$$$$$$$$$$        Sub program for HYPERGEOMETRIC RANDOM NUMBER GENERATION         $$$$$$$$$$$$$$$$$$$$$$$$$$$$$$$$$$$$$$$$

C###############################################################################################################################################
        SUBROUTINE HYPERGDIST(DHALF,DMUT,DTOT,DUMURN,IHBUSEED,XVAL)

        DOUBLE PRECISION DTOT,DHALF,DMUT,XVAL,EXPDIFF
        DOUBLE PRECISION DUMURN,SUM1,DN2,ACOUNT,ANUM,ADIN,DIFF,HBUXVAL,AMAXSAMP

        INTEGER IHBUSEED
        IF(DHALF.LT.(DTOT-DMUT))THEN
                SUM1=0.0D0
                DN2=DTOT-DMUT
                DO 322 ACOUNT=1.0D0,DN2
                        SUM1=SUM1+LOG(ACOUNT)
322             CONTINUE

                ANUM=SUM1
                SUM1=0.0D0
                DN2=DTOT-DHALF
                DO 323 ACOUNT=1.0D0,DN2
                        SUM1=SUM1+LOG(ACOUNT)
323             CONTINUE
                ANUM=ANUM+SUM1

                SUM1=0.0D0
                DN2=DTOT
                DO 324 ACOUNT=1.0D0,DN2
                        SUM1=SUM1+LOG(ACOUNT)
324             CONTINUE
                ADIN=SUM1

                SUM1=0.0D0
                DN2=(DTOT-DMUT)-DHALF
                DO 325 ACOUNT=1.0D0,DN2
                        SUM1=SUM1+LOG(ACOUNT)
325             CONTINUE
                ADIN=ADIN+SUM1
                DIFF=ANUM-ADIN
                EXPDIFF=EXP(DIFF)
                XVAL=0.0D0
        ELSE
                SUM1=0.0D0
                DN2=DMUT
                DO 326 ACOUNT=1.0D0,DN2
                        SUM1=SUM1+LOG(ACOUNT)
```

```
326             CONTINUE
                ANUM=SUM1

                SUM1=0.0D0
                DN2=DHALF
                DO 327 ACOUNT=1.0D0,DN2
                        SUM1=SUM1+LOG(ACOUNT)
327             CONTINUE
                ANUM=ANUM+SUM1

                SUM1=0.0D0
                DN2=DHALF-(DTOT-DMUT)
                DO 328 ACOUNT=1.0D0,DN2
                        SUM1=SUM1+LOG(ACOUNT)
328             CONTINUE
                ADIN=SUM1

                SUM1=0.0D0
                DN2=DTOT
                DO 329 ACOUNT=1.0D0,DN2
                        SUM1=SUM1+LOG(ACOUNT)
329             CONTINUE
                ADIN=ADIN+SUM1
                DIFF=ANUM-ADIN
                EXPDIFF=EXP(DIFF)
                XVAL=DHALF-(DTOT-DMUT)
        ENDIF

        IF(EXPDIFF.EQ.0.0D0)THEN
                CALL HBUALG(DTOT,DMUT,DHALF,IHBUSEED,HBUXVAL)
                XVAL=HBUXVAL
        ELSE
                DO WHILE (DUMURN.GT.EXPDIFF)
                        DUMURN=DUMURN-EXPDIFF
                        EXPDIFF=EXPDIFF*(((DMUT-XVAL)*(DHALF-XVAL))/((XVAL+1.0D0)*((DTOT-DMUT)-DHALF+1.0D0+XVAL)))
                        XVAL=XVAL+1.0D0
                        IF(EXPDIFF.EQ.0.0D0)THEN
                                CALL HBUALG(DTOT,DMUT,DHALF,IHBUSEED,HBUXVAL)
                                XVAL=HBUXVAL
                                GOTO 91
                        ENDIF
                ENDDO
        ENDIF

91      XVAL=XVAL

        RETURN
        END

C###############################################################################################################################

C       $$$$$$$$$$$$$$$$$$$$$$$$$$$$$$$$ Alternate program for HYPERGEOMETRIC RANDOM NUMBER GENERATION $$$$$$$$$$$$$$$$$$$$$$$$$$$$$$$$$$$$$$$$$$$$$

C###############################################################################################################################

        SUBROUTINE HBUALG(DN,DN1,DK,IALTSEED,RETHBUVAL)
        DOUBLE PRECISION DN,DN1,DN2,DK,RETHBUVAL,DT,DT1,DX,DJ,AHBUURN,TEMPTERM
```

```
        INTEGER IALTSEED,IHBUDUM,IHBUDUM2,IHBUV(32),IHBUY,IVECCOUNT

        IHBUDUM2=0
        IHBUY=0
        DO 27 IVECCOUNT=1,32
                IHBUV(IVECCOUNT)=0
27      CONTINUE

        DN2=DN-DN1
        DT=DN
        DT1=DN1

        DX=0.0D0
        DJ=0.0D0
        DO WHILE (DJ.LE.DK)
                CALL RANDOM(IALTSEED,IHBUDUM2,IHBUV,IHBUY,AHBUURN)
                TEMPTERM=DT1/DT

                IF(AHBUURN.LE.TEMPTERM)THEN
                        DX=DX+1.0D0
                        IF(DX.EQ.DN1)THEN
                                GOTO 333
                        ELSE
                                DT1=DT1-1.0D0
                        ENDIF
                ENDIF
                DT=DT-1.D0
                DJ=DJ+1.D0

        ENDDO
333     RETHBUVAL=DX

        RETURN
        END


C###########################################################################################################################################################

C       $$$$$$$$$$$$$$$$$$$$$$$$$$$$$$$$        Sub program for UNIFORM RANDOM NUMBER GENERATION        $$$$$$$$$$$$$$$$$$$$$$$$$$$$$$$$$$$$$$$$$$$$$$$

C###########################################################################################################################################################
        SUBROUTINE RANDOM(NIDUM,NIDUM2,NIV,NIY,AURN)
        INTEGER NIDUM,NIDUM2,NIV(32),NIY,IM1,IM2,IMM1,IA1,IA2,IQ1,IQ2,IR1,IR2,NTAB,NDIV,J,K
        DOUBLE PRECISION AURN,AM,EPS,RNMX
        PARAMETER(IM1=2147483563,IM2=2147483399,AM=1.0/IM1,IMM1=IM1-1,IA1=40014,IA2=40692,IQ1=53668,IQ2=52774,IR1=12211,IR2=3791,NTAB=32)
        PARAMETER(NDIV=1+IMM1/NTAB,EPS=1.2E-7,RNMX=1.0-EPS)
        IF(NIDUM.LE.0)THEN
                NIDUM=MAX(-NIDUM,1)
                NIDUM2=NIDUM
                DO 14 J=NTAB+8,1,-1
                        K=NIDUM/IQ1
                        NIDUM=(IA1*(NIDUM-(K*IQ1)))-(IR1*K)
                        IF(NIDUM.LT.0) NIDUM=NIDUM+IM1
                        IF(J.LE.NTAB) NIV(J)=NIDUM
14              CONTINUE
                NIY=NIV(1)
        ENDIF
        K=NIDUM/IQ1
```

```
      NIDUM=(IA1*(NIDUM-(K*IQ1)))-(IR1*K)
      IF(NIDUM.LT.0) NIDUM=NIDUM+IM1
      K=NIDUM2/IQ2
      NIDUM2=(IA2*(NIDUM2-(K*IQ2)))-(IR2*K)
      IF(NIDUM2.LT.0) NIDUM2=NIDUM2+IM2
      J=1+NIY/NDIV
      NIY=NIV(J)-NIDUM2
      NIV(J)=NIDUM
      IF(NIY.LT.1)NIY=NIY+IMM1
      AURN=MIN(AM*NIY,RNMX)
      RETURN
      END
```
